# Discovering protein–protein interaction stabilisers by native mass spectrometry†

**DOI:** 10.1039/d1sc01450a

**Published:** 2021-07-07

**Authors:** Jeddidiah Bellamy-Carter, Manjari Mohata, Marta Falcicchio, Jaswir Basran, Yusuke Higuchi, Richard G. Doveston, Aneika C. Leney

**Affiliations:** School of Biosciences, University of Birmingham Edgbaston Birmingham B15 2TT UK a.leney@bham.ac.uk; Leicester Institute of Structural and Chemical Biology and School of Chemistry, University of Leicester Leicester LE1 7RH UK r.g.doveston@leicester.ac.uk; Department of Molecular and Cell Biology, University of Leicester Leicester LE1 7RH UK; Department of Molecular Medicine, Beckman Research Institute of City of Hope Duarte CA 91010 USA

## Abstract

Protein–protein interactions (PPIs) are key therapeutic targets. Most PPI-targeting drugs in the clinic inhibit these important interactions; however, stabilising PPIs is an attractive alternative in cases where a PPI is disrupted in a disease state. The discovery of novel PPI stabilisers has been hindered due to the lack of tools available to monitor PPI stabilisation. Moreover, for PPI stabilisation to be detected, both the stoichiometry of binding and the shift this has on the binding equilibria need to be monitored simultaneously. Here, we show the power of native mass spectrometry (MS) in the rapid search for PPI stabilisers. To demonstrate its capability, we focussed on three PPIs between the eukaryotic regulatory protein 14-3-3σ and its binding partners estrogen receptor ERα, the tumour suppressor p53, and the kinase LRRK2, whose interactions upon the addition of a small molecule, fusicoccin A, are differentially stabilised. Within a single measurement the stoichiometry and binding equilibria between 14-3-3 and each of its binding partners was evident. Upon addition of the fusicoccin A stabiliser, a dramatic shift in binding equilibria was observed with the 14-3-3:ERα complex compared with the 14-3-3:p53 and 14-3-3:LRRK2 complexes. Our results highlight how native MS can not only distinguish the ability of stabilisers to modulate PPIs, but also give important insights into the dynamics of ternary complex formation. Finally, we show how native MS can be used as a screening tool to search for PPI stabilisers, highlighting its potential role as a primary screening technology in the hunt for novel therapeutic PPI stabilisers.

## Abstract 2

Stabilising protein–protein interactions is challenging, yet therapeutically important. Native mass spectrometry can be used to monitor binding equilibria, allowing identification and measurement of novel protein–protein interaction stabilisers.

## Graphical abstract

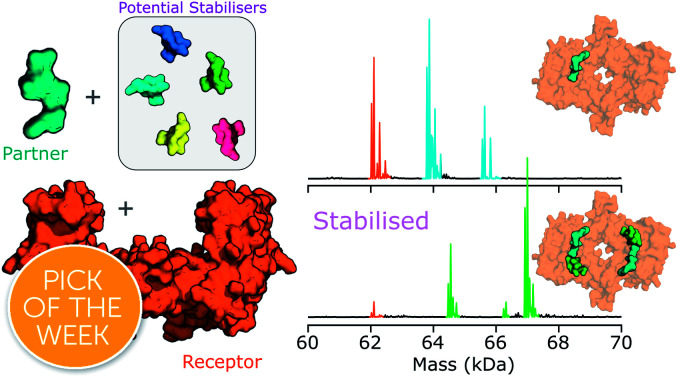

## Introduction

Stabilising protein–protein interactions (PPI) by targeting PPI interfaces with small molecule drugs has enormous potential as a therapeutic strategy. In the clinic this has been exemplified by naturally occurring compounds such as rapamycin^1^ and FK506 ^2^ (immunosuppressants), and synthetic compounds such as tafamidis^3^ (familial amyloid polyneuropathy). In contrast to PPI inhibition, however, PPI stabilisation is rarely the focus of dedicated drug development programmes. Of the few examples of small molecule PPI stabilisers, most have been discovered in a retrospective manner.^4^

**Fig. 1:**
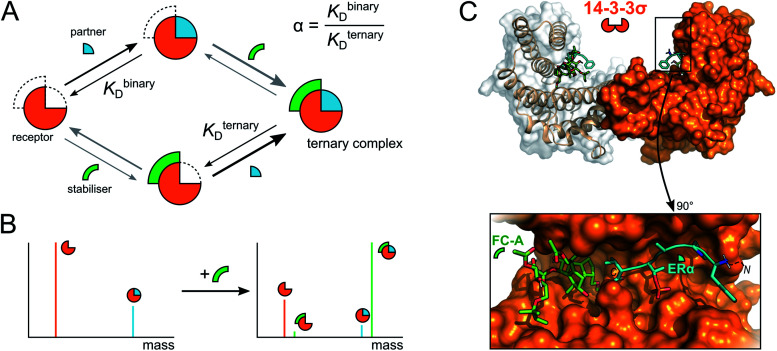
Mechanism of PPI stabilisation and anticipated mass spectral results. (A) Diagram of binding equilibria between a receptor (orange ¾ circle), its interaction partner (blue ¼ circle) and a small molecule stabiliser (green arc). Stabilisation of PPIs is often described with the quotient of the binding constants for the binary and ternary complexes (termed α).^6^ (B) Anticipated deconvoluted mass spectra for a binary receptor–partner interaction stabilised by a small molecule. (C) Cartoon rendering of the 14-3-3σ–ERα–FC-A ternary complex (PDB: 4JDD).

A significant barrier to progress in this area has been the lack of high-throughput and sensitive screening technologies capable of identifying molecular starting points for drug development.^5^ X-ray crystallography and NMR spectroscopy are powerful but relatively low-throughput techniques that lack information on binding dynamics and thus stabilisation effects. More commonly, biochemical techniques such as isothermal titration calorimetry (ITC), fluorescence-based binding assays and surface plasmon resonance (SPR) are used to measure the influence of small molecule stabilisers on the affinity of PPIs. However, each carries significant drawbacks in terms of speed, reagent consumption, or a requirement for labelling or immobilisation of one or more proteins. In addition, the binding events that underpin PPI stabilisation are complex and governed by multiple dynamic equilibria (Fig. 1a).^6^ The degree of stabilisation is typically expressed in terms of the change in affinity between the two interacting proteins (*i.e. K*_D_^binary^/*K*_D_^ternary^, Fig. 1a).^6,7^ Such analysis requires super-stoichiometric quantities of stabiliser to obtain *K*_D_^ternary^ and thus overlooks the other individual binding events. Moreover, the affinity of a stabiliser binding to individual PPI partners can be almost undetectably low, and yet it significantly stabilises the PPI *via* a cooperative mechanism.^6^ Conversely, other compounds might convey a weak or no stabilising effect, but do form protein–protein–drug ternary complexes. Such compounds would be overlooked in biochemical assays and yet are ideal starting points for optimisation into *bona fide* stabilisers.

There is therefore a genuine need for high-throughput, highly sensitive screening technologies capable of simultaneously detecting PPI stabilisation and dynamic complex formation. Here, we demonstrate that native mass spectrometry (native MS) is a powerful technique that can bridge this technology gap. Native MS, the analysis of proteins and protein complexes in their non-denatured state,^8–10^ is an incredibly versatile technique, allowing both the stoichiometry and binding equilibria of protein complexes to be determined.^11–15^ Moreover, when investigating complex mixtures with a number of potential binders, each intermediate can be observed and separately detected based on its unique mass (Fig. 1b). Coupled with its high throughput nature, this means that native MS has great potential for in-solution monitoring and screening for novel, small molecule, protein–protein interaction stabilisers. Indeed, the analysis of ternary protein–protein–ligand complexes has been elegantly demonstrated.^16^ However, this study focussed on the interaction of bifunctional proteolysis targeting chimera (ProTaC) ligands with high affinities for well-defined ligand binding pockets on both protein partners. Established biochemical techniques can therefore be used to confirm binding of the ProTaC ligand to the individual partners and provide a strong indication that ternary complex formation will be induced. This is not the case for small molecule stabilisers that act as ‘molecular glues’ and induce ternary complex formation *via* a complex cooperative effect whereby ligand binding to the individual protein partners is not detectable by conventional means. With the exception of interfacial lipids within membrane protein oligomers,^17^ to the best of our knowledge, no studies have capitalised on the potential of native MS to detect ternary complex formation in this context.

To exemplify the power of native MS in monitoring PPI stabilisation, we chose to focus on the 14-3-3 dimeric family of hub proteins. These provide an ideal platform for PPI stabiliser method development because of their significance as potential drug targets, and their detailed structural and biophysical characterisation. They play diverse and important roles in maintaining normal cell function through interaction with over 200 partner proteins.^18,19^ These PPIs are typically dependent on phosphorylation of specific recognition motifs within disordered domains of the partner protein that interact with an amphipathic groove on 14-3-3 (Fig. 1c). As a result, 14-3-3 modulates the subcellular localisation, protein folding, enzymatic activity or biomolecular interactions of the partner protein.^20^ We chose to focus on three important 14-3-3 PPIs between 14-3-3σ and LRRK2,^21^ ERα^22^ and p53 ^23,24^ which are implicated with Parkinson's disease and cancer, and which are known to be differentially stabilised by fusicoccin A (FC-A). Estrogen receptor ERα has a characteristic ‘mode 3’ C-terminal 14-3-3 binding motif that binds to 14-3-3σ, preventing ERα dimerization and thus inhibits its transcriptional activity which is a driver for breast cancer progression. FC-A stabilises this ERα–14-3-3σ interaction by 16-fold which leads to a decrease in MCF-7 cell proliferation.^22^ The tumour suppressor p53 interacts with 14-3-3σ *via* one or more phosphorylated motifs within its C-terminal domain. 14-3-3σ prevents p53 degradation by inhibiting MDM2-mediated ubiquitination, and thus stabilisation of the PPI could be an effective modality in cancer treatment. This interaction is moderately stabilised by FC-A.^23^ In contrast to p53 and ERα, leucine-rich repeat kinase 2 (LRRK2) predominantly interacts with 14-3-3 *via* its pS935 phosphorylation site on an internal domain rather than a C-terminal region; this interaction is not stabilised by FC-A.^21^ We show that the interactions between p53, ERα and LRRK2 and their interacting partner 14-3-3 can be readily detected by native MS and the stoichiometry of their interactions determined. Indeed, as predicted, native MS showed the binding equilibria of these binary interactions was shifted upon addition of FC-A. Moreover, this equilibrium shift correlated precisely with the differential stabilisation ability of FC-A in modulating the 14-3-3:ERα and 14-3-3:p53 PPIs. Finally, we show how native MS can be used as a screening tool to search for novel PPI stabilisers; a significant step forward in PPI stabilisation discovery.

## Methods

### Chemicals and reagents

Fusicoccin A (FC-A) and fusicoccin J (FC-J) were obtained as a metabolite of wildtype *Phomopsis amygdali* and genetically modified *Phomopsis amygdali* Niigata-2, as reported previously.^25^ Pyrrolidone1 (Pyr1) was synthesised according to a procedure adapted from a previous report.^26^ Full experimental details are provided in the ESI.† All other chemicals were purchased from Sigma-Aldrich unless otherwise stated. Ultrapure water (18.2 MΩ cm) and analytical grade ammonium acetate (Fisher Scientific) was used for the native MS experiments. All drug molecules were prepared in neat dimethyl sulfoxide (DMSO) as 10 mM stocks before dilution with water to the required concentration for analysis. The drug cocktail consisted of FC-A, epibestatin (Epi) (Apollo Scientific), reserpine (Res), dansyl amide (Dan) and bezafibrate (Bez); this was extended with FC-J and Pyr1. The mixture was prepared in DMSO with all drugs present at an equimolar concentration.

### Expression and purification of 14-3-3σ

Recombinant 14-3-3σ with a TEV protease cleavable N-terminal His-tag was expressed in BL21 (DE3) competent cells with a pPROEX Htb plasmid, and purified by Ni^2+^ affinity chromatography, sequence in ESI.† The protein was dialysed against buffer containing 25 mM HEPES, 100 mM NaCl, 10 mM MgCl_2_, 2 mM β-mercaptoethanol, pH 8.0. The protein was then further purified by size-exclusion chromatography using a Superdex 75 column, eluting with the same buffer. Protein-containing fractions were concentrated to 0.72 mM using a centrifugal filter unit (Merck Millipore).

### Generation of phosphopeptides

Phosphopeptides were used to mimic 14-3-3σ binding partners. Thus, the C-terminal fragments of p53 (Ac-RHKKLMFK(pT)EGPDSD–COOH) and ERα (Ac-KYYITGEAEGFPA(pT)V–COOH) and an internal fragment of LRRK2 (Ac-NLQRHSN(pS)LGPIFDH–CONH_2_) were synthesised commercially corresponding to p53_379–393_, ERα_581–595_ and LRRK2_928–942_. The peptides were purchased at >95% purity from Synpeptide Ltd (ERα and LRRK2 peptides) or ChinaPeptides (p53). The lyophilised peptides were reconstituted to 1 mM in 50 mM ammonium acetate (pH 6.8) and stored at −20 °C until further use.

### Preparation of proteins for native mass spectrometry

Purified recombinant 14-3-3σ was exchanged into 100 mM ammonium acetate (pH 6.8) using an Amicon Ultra 0.5 mL centrifugal concentrator (Merck Millipore) with successive dilutions and concentrations. The exchanged protein was stored at −20 °C until use. A working stock of 20 μM (monomer) was diluted immediately before native MS analysis. Lyophilised myoglobin (horse heart) was purchased from Sigma-Aldrich, diluted to a stock concentration of 60 μM in 100 mM ammonium acetate (pH 6.8) and stored at 4 °C prior to use.

### Native mass spectrometry

Buffer exchanged 14-3-3σ was diluted to a final concentration of 5 μM (monomer) with and without the addition of peptides and the corresponding drug of interest. For all binding experiments, a final concentration of 50 mM ammonium acetate pH 6.8 and 0.25% DMSO was used. For the drug cocktail alone, a drug concentration of 5 μM each was used in 50 mM ammonium acetate, 0.25% DMSO. Phosphopeptides were added at a 5 or 1 molar ratio to the 14-3-3σ (monomer). Drug compounds were added at a 1 or 5 molar ratio to the 14-3-3σ (monomer). For the drug binding experiments, the DMSO containing components were added to the peptides prior to 14-3-3σ addition. To control for non-specific binding,^27^ Myoglobin (equine heart) was mixed with each of the individual peptides in the presence or absence of the drug cocktail at the same molar ratios as the 14-3-3σ experiments. While small amounts of non-specific binding of LRRK2 (∼4%) was observed, p53 and ERα showed negligible binding to myoglobin (Fig. S10†). No binding was observed between the drug cocktail and myoglobin (Fig. S11†). All MS experiments were performed on a Q-Exactive HF instrument (Thermo Fisher Scientific); typically coupled to a Triversa NanoMate (Advion) to introduce samples by nanoelectrospray ionisation. Positive ionisation mode was used with a voltage of 1.75 kV and a gas pressure of 0.3 psi applied. The source temperature was set at 250 °C, in-source dissociation off, S-lens RF at 100 and a mass range of 1000–6000 *m*/*z* used to monitor the binding equilibrium. Mass spectra were scanned with a maximum ion injection time set to 100 ms, automatic gain control of 1 × 10^6^ and resolution of 15 000. An observed +76 Da adduct was attributed to partial β-mercaptoethanol capping of Cys38, see ESI and Fig. S2† for details.

### Data processing

All spectra were processed initially with Xcalibur 4.3 before deconvolution either manually or using UniDec 4.2.2,^28^ see ESI† for details. The saturation coefficient, *S*_P_, of a binding partner (P) to the receptor 14-3-3 (R) was calculated using eqn (1a) from the relative abundances (Ab_*n*,*i*_, see eqn (1b)) of all stoichiometries of *R* × *n*P × *i*L, where *n* is the number of P molecules bound and *i* is the number of ligand (L) molecules bound. The relative abundances were calculated from the peak integrals of deconvoluted mass spectra using UniDec, with only the values for the dimeric 14-3-3 species considered. This saturation coefficient represents the binding equilibrium between the PPIs. Assuming the binding sites are independent but identical, the *K*_D_ for each is the same and *S*_P_ is related to *K*_D_ by eqn (2), where [*P*]_bound_ is the concentration of peptide bound to the receptor and [*R*]_0_ is the initial concentration of the receptor (for the dimer in the case of 14-3-3σ where *n* = 2).1a
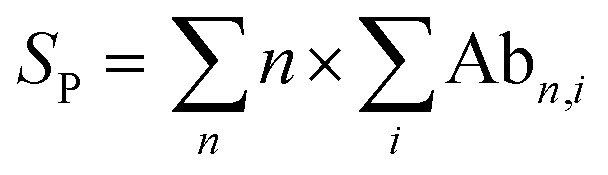
1b
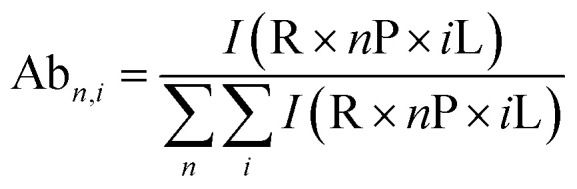
2
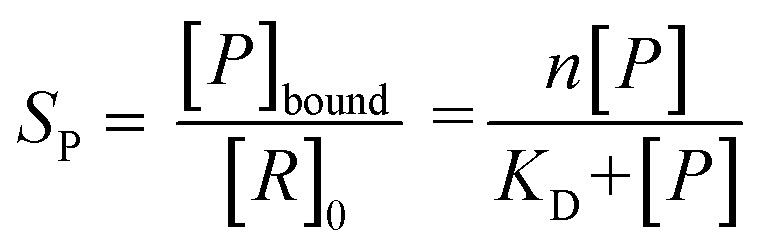


## Results and discussion

### Probing binary complex formation between 14-3-3σ and its interaction partners

**Fig. 2:**
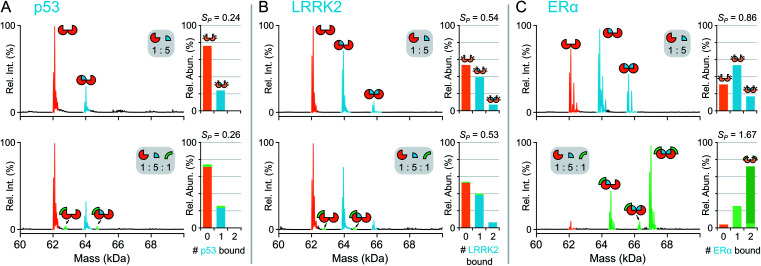
Native MS of 14-3-3σ binary and ternary complexes. Deconvoluted mass spectra for binding of p53 (A), LRRK2 (B) and ERα (C). Concentrations were 5 μM 14-3-3σ (monomer), 25 μM interaction partner and 5 μM FC-A. Binding stoichiometry is indicated with geometric representations. Inset stacked bar graphs show stoichiometry of peptide molecules (0, 1 or 2) bound to 14-3-3σ dimer, the number of FC-A molecules is indicated in the stacks with light or dark green for 1 or 2 molecules, respectively. See Fig. S4–S6† for raw and deconvoluted spectra.

First, the complex stoichiometry of 14-3-3σ was probed using native MS. Consistent with previous crystallographic studies,^22^ 14-3-3σ was observed predominantly as a dimer (Fig. S1†) indicating that complex organisation was preserved during native MS analysis and 14-3-3σ was detected in its functionally relevant state. Next, 14-3-3σ was incubated separately with three different binding partners; p53_379–393_, LRRK2_928–942_ and ERα_581–595_ (termed p53, LRRK2, and ERα hereafter). Consistent with their biological roles, p53, LRRK2 and ERα were all detected bound to dimeric 14-3-3σ (Fig. 2). The extent of partner binding to 14-3-3σ was derived from the relative abundances of all stoichiometric combinations, and expressed in terms of a saturation coefficient, *S*_P_ (eqn (1)). p53 bound the least whereby with a five-fold excess of p53, only one binding site on 14-3-3σ was occupied (*S*_P_ = 0.24, Fig. 2a, Table 1) reflective of its low binding affinity in solution. In contrast, with the same concentration ratio, LRRK2 bound both binding sites of the 14-3-3σ binding groove simultaneously (*S*_P_ = 0.54, Fig. 2b, Table 1), consistent with its known higher affinity measured using ITC.^21,23^ Even greater apparent binding was observed for ERα (*S*_P_ = 0.86, Fig. 2c, Table 1), with both binding sites partially occupied, again consistent with the higher affinity of ERα compared with LRRK2 in solution.^22^ This snapshot of binding equilibria is essential for monitoring PPI stabilisation. Moreover, a successful PPI stabiliser causes a large shift in binding equilibria, which is only possible to detect if individual binding events can be captured and uniquely identified.

**Table 1:** Comparison of native MS data for binary and ternary complex formation

Partner (P)	Ligand (L)	Ratio (R : P : L)	Bound 14-3-3σ	Saturation^a^ (*S*_P_)
ERα	—	1 : 5 : 0	70%	0.86
	FC-A	1 : 5 : 1	96%	1.67
	FC-A	1 : 5 : 5	100%	2.00
	Cocktail	1 : 5 : 1	95%	1.61
	—	1 : 1 : 0	9.4%	0.09
	FC-A	1 : 1 : 1	18%	0.21

LRRK2	—	1 : 5 : 0	47%	0.54
	FC-A	1 : 5 : 1	46%	0.53

p53	—	1 : 5 : 0	24%	0.24
	FC-A	1 : 5 : 1	26%	0.26
	—	1 : 20 : 0	68%	0.90
	FC-A	1 : 20 : 5	67%	0.95

a Values were derived from the native MS data using eqn (1)–(2).

### Probing binary and ternary complexes of 14-3-3σ, its interaction partners and FC-A

To confirm that native MS could be used to monitor protein–protein interaction stabilisation, fusicoccin A (FC-A), was incubated with the binary complexes to see if the equilibria between the PPIs was perturbed. Both p53 and ERα are known to have PPIs with 14-3-3σ that are stabilised by FC-A with relatively low^23^ and high^22^ potency, respectively. Thus, upon addition of FC-A, one would expect more higher order complexes to be observed. In contrast, while LRRK2 is a known binder of 14-3-3σ, its binding properties have shown to be independent of FC-A binding,^21^ thus no change in binding equilibria between the 14-3-3σ and LRRK2 complexes would be observed upon addition of FC-A. Upon addition of FC-A to 14-3-3σ and p53 (1 : 5 : 1 ratio of 14-3-3 : p53 : FC-A), a ternary complex was observed between the 14-3-3σ dimer, p53 and FC-A (Fig. 2a). Upon quantifying complex formation, more binding was observed between p53 and 14-3-3σ in the presence of FC-A, although to a modest degree (*S*_P_ = 0.26, Table 1). Further increasing the FC-A concentration to a ratio of 1 : 5 : 5 (14-3-3σ : p53 : FC-A) showed little additional stabilisation (Fig. S4†). However, upon addition of a 20-fold excess of p53 compared with 14-3-3σ whilst maintaining FC-A in a 5-fold excess, a clear shift in the binding of the FC-A itself to the 14-3-3σ–p53 complexes was observed (Fig. S5†). Indeed, although modest stabilisation is observed at these concentration ratios, *S*_P_ increase of 0.05, the affinity of FC-A for 14-3-3σ is clearly dependent upon occupancy of the p53 binding sites.

In stark contrast, a dramatic shift in binding equilibria was observed between 14-3-3σ and ERα upon addition of FC-A, where the predominant peaks in the mass spectrum corresponded to a (14-3-3σ)_2_–(ERα)_2_–(FC-A)_2_ ternary complex (Fig. 2c and S6†); a change in *S*_P_ from 0.86 to 1.67 corresponding to a 94% increase (Table 1). Moreover, the absence of 14-3-3σ–ERα complexes without FC-A bound is striking, more so when considering the prominence of the (14-3-3σ)_2_–(ERα)_2_–(FC-A)_2_ species. It should be noted that no ERα–FC-A complexes were observed and that the 14-3-3σ–FC-A complex is negligible—FC-A almost exclusively binds to the binary 14-3-3σ–ERα complex. Importantly, no binding stoichiometry beyond the complete ternary complex was observed, showing that the observations are indeed physiologically relevant and match those predicted based on crystallographic studies.^22,23^ It is not until now, however, that the stoichiometry of binding and degree of stabilisation has been measured simultaneously, showing the power of native MS in monitoring these stabilising interactions. Finally, as a negative control, FC-A was incubated together with 14-3-3σ and LRRK2. As expected, <1% binding between the 14-3-3σ–LRRK2 binary complex and FC-A was observed, consistent with FC-A's non-stabilising effect on LRRK2 binding to 14-3-3σ (Fig. 2b and S7†).^21^

**Fig. 3:**
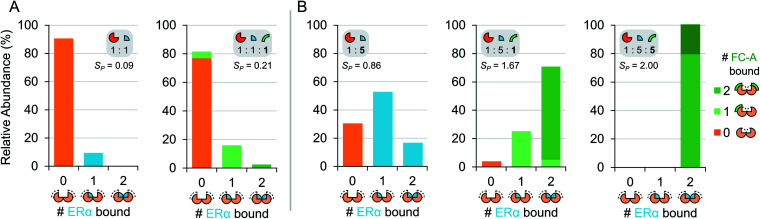
Titration of ERα and FC-A in ternary mixture with 14-3-3σ. Relative abundance stacked bar graphs showing stoichiometry of ERα molecules (0, 1 or 2) and FC-A molecules (0, 1 or >2) bound to 14-3-3σ dimer under different mixing conditions: (A) equimolar ERα to 14-3-3σ (5 μM) and (B) 5-fold excess of ERα to 14-3-3σ. Number of bound FC-A molecules is indicated in the stacks with light or dark green for 1 or 2 molecules, respectively. Note: the stabilisation of FC-A is concentration dependent. See Fig. S6† for raw and deconvoluted spectra.

To gain further insight into the mode of cooperativity between FC-A and ERα binding to 14-3-3σ, various ERα and FC-A concentrations were mixed with 14-3-3σ (Fig. 3 and S6†). As expected, when the concentration of ERα increases, more binding to 14-3-3σ is observed (Fig. 3b, Table 1). Similarly, as the concentration of FC-A increases, the level of saturation increases to complete saturation (Fig. 3b, Table 1). Curiously, the extent of FC-A stabilisation was found to be ERα concentration dependent. As already established, the addition of FC-A (1 molar equivalent) to a 1 : 5 mixture of 14-3-3σ:ERα results in a significant shift in the stoichiometry towards higher saturation (*S*_P_ 0.86 to 1.67, Fig. 2c, Table 1). However, addition of 1 molar equivalent of FC-A to a 1 : 1 mixture of 14-3-3σ:ERα leads to a much-reduced stoichiometric shift, with a change in *S*_P_ from 0.09 to 0.21 (Fig. 3, Table 1). Even at this lower concentration of ERα, the absolute binding of FC-A is evident; the interaction of ERα and FC-A in the 14-3-3σ binding pocket is highly stable.

This observation is highly significant because it indicates that FC-A potency is dependent on the extent of 14-3-3σ–ERα binary complex formation. Put another way, the effect of FC-A is much diminished in an environment where apo-14-3-3σ protein is the predominant component. The data suggests that FC-A preferentially binds to the 14-3-3σ–ERα binary complex, and thus perturbs the dynamic equilibrium to a much greater degree under these conditions. Observations like this frequently go undetected using other biochemical techniques. It further highlights the power of native MS for monitoring subtle variations in co-operative and dynamic systems.

### Screening for stabilisers in a drug cocktail

**Fig. 4:**
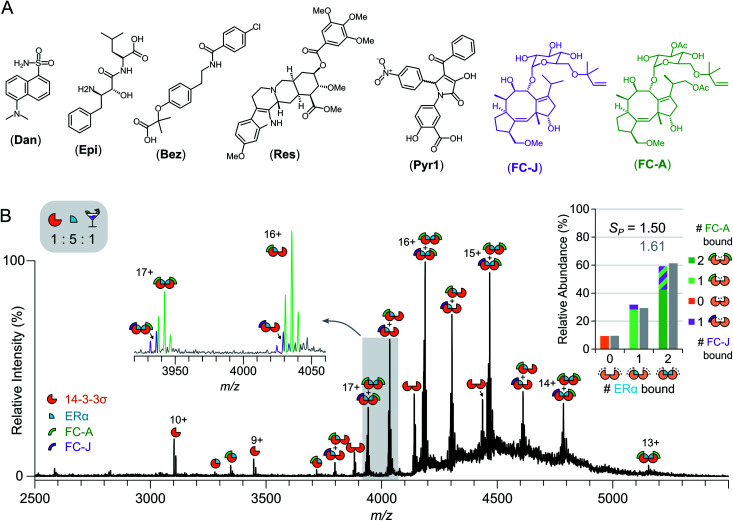
Drug cocktail screening against 14-3-3σ. (A) Chemical structures of five drugs used in drug cocktail. (B) Native mass spectrum of 14-3-3σ (5 μM) mixed with ERα (25 μM) and the drug cocktail (FC-A; epibestatin, Epi; reserpine, Res; bezafibrate, Bez; dansyl amide, Dan; pyrrolidone 1, Pyr1; fusicoccin J, FC-J; 5 μM each) shows both FC-A and FC-J bind and stabilise the PPI to different extents. Inset spectrum shows close-up of 3920–4060 *m*/*z* region. Peaks are annotated with geometric representations showing stoichiometry; charge states are shown for fully saturated 14-3-3σ species (monomer and dimer). Inset stacked bar chart shows the relative abundance of different stoichiometries of ERα binding (0, 1 or 2 molecules) to 14-3-3σ dimer, stacks split by stoichiometry of FC-A (green) or FC-J (purple) binding (0, 1 or 2 molecules); broad integration (±400 Da) around ERα stoichiometries are shown in grey.

The relative ease with which native MS can be used to detect stabilisation of 14-3-3σ–protein interactions, and ternary complex formation, opens up the possibility of high-throughput screening for the detection of PPI stabilisers. To demonstrate this, a cocktail of drugs was constructed containing both putative stabilisers FC-A and epibestatin,^29^ in addition to putative non-binders: reserpine, dansyl amide and bezafibrate (Fig. 4a and S12†). If a drug stabilised the 14-3-3σ–partner complex, as a result of ternary complex formation (or another mechanism), then the saturation coefficient, *S*_P_, would increase (Fig. 1). If a drug interacted with a 14-3-3σ–partner binary complex (or either individual component), but did not stabilise the interaction, although a ternary complex would be observed, the saturation coefficient, *S*_P_, would remain constant. Thus, native MS could distinguish, in a single experiment, between binders to 14-3-3σ, binders to 14-3-3σ's interaction partners, binders to the 14-3-3σ–partner binary complexes and 14-3-3σ–partner interaction stabilisers. To test this hypothesis, we incubated 14-3-3σ with ERα and the drug cocktail (Fig. S8†). The stoichiometry of the 14-3-3σ–ERα complexes shifted to resemble the FC-A only experiments (Fig. 2c), where the predominant peaks in the mass spectrum corresponded to a ((14-3-3σ)_2_–(ERα)_2_–(FC-A)_2_) ternary complex. This was characterised by a change in saturation, *S*_P_, from 0.86 to 1.61 (Table 1). No other peaks corresponding to ternary complexes were observed, suggesting that FC-A is the only drug within the cocktail that binds to, and stabilises, the 14-3-3σ–ERα complex. In addition, none of the drugs were found to bind to the 14-3-3σ–partner complexes for p53 and LRRK2 (Fig. S8†). Interestingly, epibestatin, was not observed to bind to the 14-3-3σ–ERα complexes, even when mixed in the absence of the other drugs (Fig. S9†). Epibestatin is not explicitly known to stabilise any of the PPIs studied here; however, it has been shown to stabilise the interaction of 14-3-3ε with the plant proton pump PMA2, a PPI also stabilised by FC-A to a significant extent.^29^ The other drugs in the cocktail also showed a lack of binding when incubated independently with 14-3-3σ:ERα (Fig. S9†). To test this methodology further, two additional putative stabilisers were introduced: fusicoccin J (FC-J), a biosynthetic precursor to FC-A;^25^ and pyrrolidone 1 (Pyr1), a weak stabiliser of several 14-3-3–partner interactions.^26,29,30^ On their own, like with FC-A, FC-J binds more strongly to 14-3-3σ–ERα complexes albeit to a lesser extent with only moderate stabilisation (*S*_P_ = 0.96, Fig. S13†). Pyr1, on the other hand, shows negligible stabilisation for 14-3-3σ–ERα but does bind to both 14-3-3σ and the 14-3-3σ–ERα complexes (Fig. S13†). Extending the previous cocktail with these two drugs (Fig. 4a) highlights further the advantages of native MS (Fig. 4b and S14†). In the native mass spectrum, both FC-A and FC-J are observed in a ternary complex with 14-3-3σ and ERα (*S*_P_ = 1.50). Indeed, all 14-3-3σ–ERα complexes have at least one molecule of FC-A or FC-J bound. The (14-3-3σ)_2_–(ERα)_1_ complex has either FC-A or FC-J bound but not both, suggesting that both FCs have a similar binding mechanism and compete for the same site. Most excitingly, the (14-3-3σ)_2_–(ERα)_2_ complex is observed in two stoichiometries: (FC-A)_2_, (FC-A)(FC-J). That is to say that not only can native MS be used to detect stabilisers from a mixture, as shown here, it can observe complexes of multiple stabilisers to the same protein–protein interaction, something not possible through SPR or ITC. Negligible binding of Pyr1 was observed from this extended cocktail. This was not unexpected due to the higher affinity and greater stabilisation ability of the FC-A and FC-J within the cocktail. Thus, native MS can serve as an enlightening tool, particularly as the ‘best’ stabilisers and binders will win out. Thus, PPI stabilisers can indeed be identified from drug cocktails with relative ease *via* native MS; opening the door for larger scale screening of putative PPI stabilisers.

## Conclusions

Native MS is accelerating in its applications in monitoring PPIs within the pharmaceutical industry. To date, methodology has focused on detecting PPI inhibitors. Stabilising PPI interactions offers a promising therapeutic alternative, yet is more challenging to monitor since both the stoichiometry of binary and ternary complexes, and knowledge on how the equilibrium between these complexes shifts upon addition of the potential stabiliser is required. Here, we show how native MS can distinguish, in a single experiment, between binders to either protein in the PPI, binders to the protein–protein complex that do not undergo stabilisation, and binders to the protein–protein complex that stabilise/enhance the formation of the PPI.

We highlighted this on a dimeric hub protein 14-3-3σ, which is of therapeutic interest due to its role in binding with hundreds of proteins within cells; the interactions of which are mis-regulated in cancer and neurodegenerative disease. Our data showed that native MS can differentiate the ability of FC-A in stabilising the 14-3-3:ERα and 14-3-3:p53 complexes. In addition, native MS was also able to verify PPIs whereby FC-A did not stabilise the PPI, such as the 14-3-3:LRRK2 interaction. Finally, we showed how stabilisers could be picked out rapidly from within a drug cocktail; showing the potential of native MS for high throughput screening of novel PPI stabilisers. Additionally, it is important to note that native MS is not restricted to monitor only PPI stabilisers in a screening approach—it is possible to simultaneously detect stabilisers and inhibitors of PPIs using this technology. Thus, we anticipate that the methods described herein will be applied to a variety of clinically relevant PPIs and will become an integral part of the toolbox alongside SPR and ITC, acting in the first line of inquiry for the discovery of PPI modulators.

## Data availability

Raw data files supporting this research is openly available from the University of Birmingham data archive at https://doi.org/10.25500/eData.bham.00000673.

## Author contributions

Native mass spectrometry investigations were performed by JBC and MM. Recombinant 14-3-3σ was cloned and expressed by JB and RGD. FC-J was provided by YH. Pyr1 was synthesised by MF and RGD. Conceptualisation by ACL and RGD. The manuscript was written and revised by JBC, ACL and RGD.

## Conflicts of interest

There are no conflicts to declare.

## Supplementary Material

SC-012-D1SC01450A-s001

## References

[cit1] Brown E. J., Albers M. W., Bum Shin T., Ichikawa K., Keith C. T., Lane W. S., Schreiber S. L. (1994). Nature.

[cit2] Griffith J. P., Kim J. L., Kim E. E., Sintchak M. D., Thomson J. A., Fitzgibbon M. J., Fleming M. A., Caron P. R., Hsiao K., Navia M. A. (1995). Cell.

[cit3] Lamb Y. N., Deeks E. D. (2019). Drugs.

[cit4] Andrei S. A., Sijbesma E., Hann M., Davis J., O'Mahony G., Perry M. W. D., Karawajczyk A., Eickhoff J., Brunsveld L., Doveston R. G., Milroy L.-G., Ottmann C. (2017). Expert Opin. Drug Discovery.

[cit5] Milroy L.-G., Grossmann T. N., Hennig S., Brunsveld L., Ottmann C. (2014). Chem. Rev..

[cit6] de Vink P. J., Andrei S. A., Higuchi Y., Ottmann C., Milroy L.-G., Brunsveld L. (2019). Chem. Sci..

[cit7] Douglass E. F., Miller C. J., Sparer G., Shapiro H., Spiegel D. A. (2013). J. Am. Chem. Soc..

[cit8] Loo J. A. (1997). Mass Spectrom. Rev..

[cit9] Leney A. C., Heck A. J. R. (2017). J. Am. Soc. Mass Spectrom..

[cit10] Benesch J. L. P., Ruotolo B. T., Simmons D. A., Robinson C. V. (2007). Chem. Rev..

[cit11] Hernández H., Robinson C. V. (2007). Nat. Protoc..

[cit12] Ebong I.-o., Morgner N., Zhou M., Saraiva M. A., Daturpalli S., Jackson S. E., Robinson C. V. (2011). Proc. Natl. Acad. Sci. U. S. A..

[cit13] Kitova E. N., Seo M., Roy P.-N., Klassen J. S. (2008). J. Am. Chem. Soc..

[cit14] Kitova E. N., El-Hawiet A., Schnier P. D., Klassen J. S. (2012). J. Am. Soc. Mass Spectrom..

[cit15] Cubrilovic D., Biela A., Sielaff F., Steinmetzer T., Klebe G., Zenobi R. (2012). J. Am. Soc. Mass Spectrom..

[cit16] Beveridge R., Kessler D., Rumpel K., Ettmayer P., Meinhart A., Clausen T. (2020). ACS Cent. Sci..

[cit17] Gupta K., Donlan J. A. C., Hopper J. T. S., Uzdavinys P., Landreh M., Struwe W. B., Drew D., Baldwin A. J., Stansfeld P. J., Robinson C. V. (2017). Nature.

[cit18] Johnson C., Crowther S., Stafford M. J., Campbell D. G., Toth R., MacKintosh C. (2010). Biochem. J..

[cit19] Stevers L. M., Sijbesma E., Botta M., MacKintosh C., Obsil T., Landrieu I., Cau Y., Wilson A. J., Karawajczyk A., Eickhoff J., Davis J., Hann M., O'Mahony G., Doveston R. G., Brunsveld L., Ottmann C. (2018). J. Med. Chem..

[cit20] Pennington K. L., Chan T. Y., Torres M. P., Andersen J. L. (2018). Oncogene.

[cit21] Stevers L. M., de Vries R. M. J. M., Doveston R. G., Milroy L.-G., Brunsveld L., Ottmann C. (2017). Biochem. J..

[cit22] Leeuwen I. J. D. V., Pereira D. da C., Flach K. D., Piersma S. R., Haase C., Bier D., Yalcin Z., Michalides R., Feenstra K. A., Jiménez C. R., de Greef T. F. A., Brunsveld L., Ottmann C., Zwart W., de Boer A. H. (2013). Proc. Natl. Acad. Sci. U. S. A..

[cit23] Doveston R. G., Kuusk A., Andrei S. A., Leysen S., Cao Q., Castaldi M. P., Hendricks A., Brunsveld L., Chen H., Boyd H., Ottmann C. (2017). FEBS Lett..

[cit24] Falcicchio M., Ward J. A., Macip S., Doveston R. G. (2020). Cell Death Discovery.

[cit25] Noike M., Ono Y., Araki Y., Tanio R., Higuchi Y., Nitta H., Hamano Y., Toyomasu T., Sassa T., Kato N., Dairi T. (2012). PLoS One.

[cit26] Richter A., Rose R., Hedberg C., Waldmann H., Ottmann C. (2012). Chem.–Eur. J..

[cit27] Sun J., Kitova E. N., Wang W., Klassen J. S. (2006). Anal. Chem..

[cit28] Marty M. T., Baldwin A. J., Marklund E. G., Hochberg G. K. A., Benesch J. L. P., Robinson C. V. (2015). Anal. Chem..

[cit29] Rose R., Erdmann S., Bovens S., Wolf A., Rose M., Hennig S., Waldmann H., Ottmann C. (2010). Angew. Chem., Int. Ed..

[cit30] Bosica F., Andrei S. A., Neves J. F., Brandt P., Gunnarsson A., Landrieu I., Ottmann C., O'Mahony G. (2020). Chem.–Eur. J..

