# Supplementary material for: Discovering protein–protein interaction stabilisers by native mass spectrometry
Source: Chem Sci. 2021 Jul 7;12(32):10724–31. doi: 10.1039/d1sc01450a (PMC8372317; doi:10.1039/d1sc01450a)
Supplement: SC-012-D1SC01450A-s001 [file SC-012-D1SC01450A-s001.pdf]

Electronic Supplementary Material (ESI) for Chemical Science.
This journal is © The Royal Society of Chemistry 2021

Supporting Information

Discovering Protein–Protein Interaction Stabilisers by Native Mass Spectrometry

Jeddidiah Bellamy-Carter,[a] Manjari Mohata,[a] Marta Falcicchio,[b] Jaswir Basran,[c] Yusuke Higuchi,[d] Richard G. Doveston[b]*, Aneika C. Leney[a]*

[a] School of Biosciences, University of Birmingham, Edgbaston, Birmingham, B15 2TT, UK.

[b] Institute of Structural and Chemical Biology and School of Chemistry, University of Leicester, Leicester, LE1 7RH, UK.

[c] Department of Molecular and Cell Biology, University of Leicester, Leicester, LE1 7RH, UK.

[d] Department of Molecular Medicine, Beckman Research Institute of City of Hope, Duarte, CA 91010, USA

*Correspondence should be addressed to: *a.leney@bham.ac.uk and r.g.doveston@leicester.ac.uk*

Contents

Supplementary Methods **2**
- Characterisation of 14-3-3σ native MS 2
- Tryptic digestion of 14-3-3σ 2
- Deconvolution of native MS 2

Supplementary Figures **3**
- Characterisation of 14-3-3σ by native mass spectrometry 3
- Binding of FC-A to 14-3-3σ protein complexes 4
- Binding of drug cocktail to 14-3-3σ protein complexes 9
- Nonspecific binding of phosphopeptides and drug compounds 11
- Screening extended drug cocktail for 14-3-3σ–ERα stabilisers 13

References **19**

List of Figures

Figure S1 | Native MS spectrum of 14-3-3σ. 3
Figure S2 | MS/MS spectra identifying β-mercaptoethanol-modification on Cys38 of 14-3-3σ. 3
Figure S3 | Native mass spectra of FC-A binding to 14-3-3σ at 1:1 and 1:5 ratios. 4
Figure S4 | Native mass spectra of 14-3-3σ, p53 and FC-A at different ratios. 5
Figure S5 | Native mass spectra of 14-3-3σ, p53 and FC-A at significant excess. 6
Figure S6 | Native mass spectra of 14-3-3σ, ERα and FC-A at different ratios. 7
Figure S7 | Native mass spectra of 14-3-3σ, LRRK2 and FC-A at different ratios. 8
Figure S8 | Differential binding of drug cocktail to 14-3-3σ protein complexes with p53, LRRK2 and ERα. 9
Figure S9 | Negligible binding of drugs: Epi, Res, Bez and Dan to 14-3-3σ–ERα protein complexes at a 1:5:1 ratio. 10
Figure S10 | Negligible non-specific binding of p53, LRRK2 and ERα to myoglobin. 11
Figure S11 | Negligible non-specific binding of p53, LRRK2 and ERα, and drug cocktail to myoglobin. 11
Figure S12 | Spectra of drug cocktail (Dan, Epi, Bez, Res, FC-A). 12
Figure S13 | Native mass spectra of 14-3-3σ and ERα binding to FC-J or Pyr1. 13
Figure S14 | Extended cocktail binding to 14-3-3σ and ERα. 14
Scheme S1 | Synthetic route to Pyr1. 16
Figure S15 | Spectroscopic characterisation of Pyr1. 18

List of Tables

Table S1 | Molecular weight assignments for protein, peptides and drug compounds. 15

Supplementary Methods

Amino acid sequence for recombinant 14-3-3σ (numbered from native start methionine), recombinant purification tag residues are shown in lowercase:

```
-28         -18         -8          3           13          23
msyyhhhhhh  dydipttenl  yfqgamgsME  RASLIQKAKL  AEQAERYEDM  AAFMKGAVEK
33          43          53          63          73          83
GEELSCEERN  LLSVAYKNVV  GGQRAAWRVL  SSIEQKSNEE  GSEEKGPEVR  EYREKVETEL
93          103         113         123         133         143
QGVCDTVLGL  LDSHLIKEAG  DAESRVFYLK  MKGDYYRYLA  EVATGDDKKR  IIDSARSAYQ
153         163         173         183         193         203
EAMDISKKEM  PPTNPIRLGL  ALNFSVFHYE  IANSPEEAIS  LAKTTFDEAM  ADLHTLSEDS
213         223         233         243
YKDSTLIMQL  LRDNLTLWTA  DNAGEEGGEA  PQEPQS
```

Characterisation of 14-3-3σ native MS

Native MS of the recombinant 14-3-3σ protein showed predominantly dimer (~62 kDa, Figure S1). The monomeric 14-3-3σ was measured with two isoforms: at 31013 ± 0.8 and 31089 ± 1.2 Da in approximately equal amounts, corresponding to loss of the N-terminal methionine (theoretical average mass of 31014.5 Da) and an additional +76 Da modification (suspected to be β-mercaptoethanol capping of a cysteine), respectively. These monomer isoforms produced three dimer mass variants (two homodimeric and one heterodimeric) at 62026 ± 0.4 Da, 62102 ± 0.5 Da and 62174 ± 0.7 Da, with the 62102 Da heterodimer being the most abundant.

Tryptic digestion of 14-3-3σ

To confirm the identity and location of the +76 Da modification, 14-3-3σ (2 µM final) was digested with trypsin (10 ng/µL final) in 100 mM ammonium bicarbonate for 2 hours at 37°C. The digest reaction was quenched with formic acid (0.2% final) and analysed by LC–MS/MS with a linear gradient (4–55% acetonitrile) on a PepMap100 C_{18} nanoViper analytical column (Thermo Fisher Scientific). Raw data was database searched using SearchGUI (v3.3.21) with X!Tandem (v2015.12.15.2) and Andromeda (v1.5.3.4) search engines on a modified cRAP FASTA database (ftp://ftp.thegpm.org/fasta/cRAP) containing the recombinant 14-3-3σ sequence. The β-mercaptoethanol adduct (Unimod accession #303) was added as a variable modification for cysteine residues. Search results were processed in PeptideShaker (v1.16.45) and peptides with both modified and unmodified PSMs compared, confirming β-mercaptoethanol adduction to Cys38. Figure S2 shows the MS/MS spectra for one such peptide (^{33}GEELS**C**EER^{41}).

Deconvolution of native MS

Spectra were deconvoluted using UniDec (v4.2.2). Briefly, a combined mass spectrum was extracted using Xcalibur (v4.1) and saved to a text document representing the single combined mass spectrum for the experiment. This text file was imported into UniDec GUI and processed as follows: mass range of 2000–6000 *m/z* with background subtraction under default settings. Deconvolution settings were: mass range of 20000–100000 Da, sampling window of 1 Da, no suppression of artefacts, automatic peak width. Peak picking was done with a window of 20 Da, threshold of 0.01. Peaks were then filtered to remove peaks with a DScore < 35. Zero-charge mass spectrum peaks were assigned using the Oligomer Mass Tool with masses for both 14-3-3σ monomer variants (31013 and 31089 Da), the peptide average mass and the average mass of the drugs (see Table S1), according to the experiment mixture. Peak integrals (with the 20 Da window) were used for calculation of relative abundance. For simplicity, only complexes of peptide/drug to the heterodimer (62102 Da) were used for relative abundance calculations.

Supplementary Figures

Characterisation of 14-3-3σ by native mass spectrometry

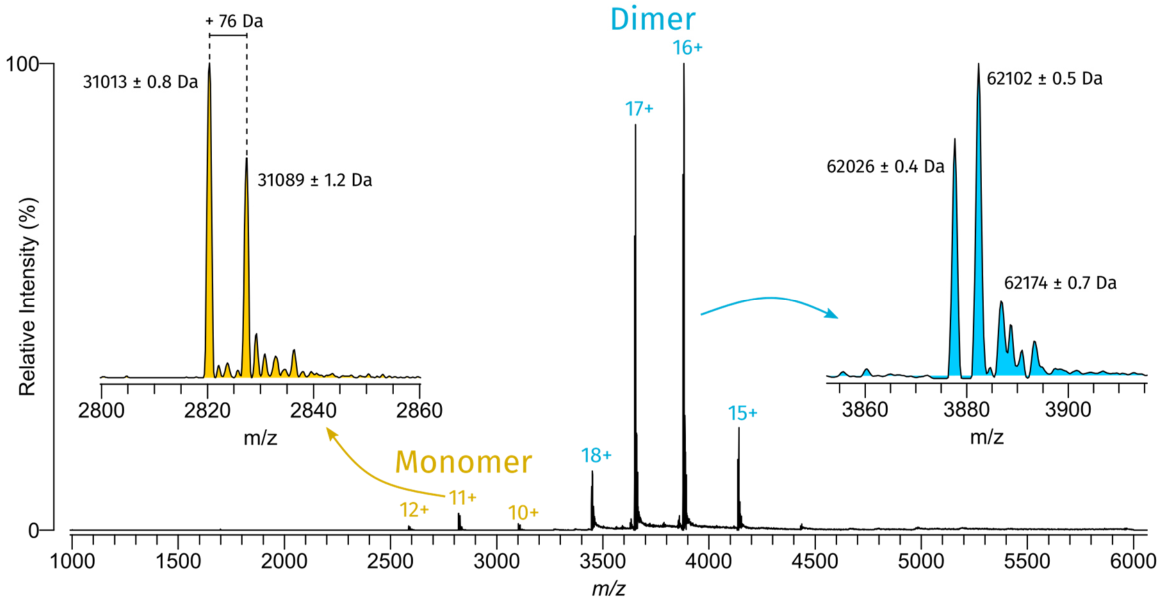

Figure S1 | Native MS spectrum of 14-3-3σ. Sprayed from 50 mM ammonium acetate. Monomer and dimer charge states are labelled. Insets show the 11+ monomer and 16+ dimer regions with the observed species labelled with deconvoluted masses (calculated from the 10+, 11+ and 12+ ions for the monomer and the 15+, 16+, 17+ and 18+ ions for the dimer species). The error is shown represents the standard deviation calculated across all charge states.

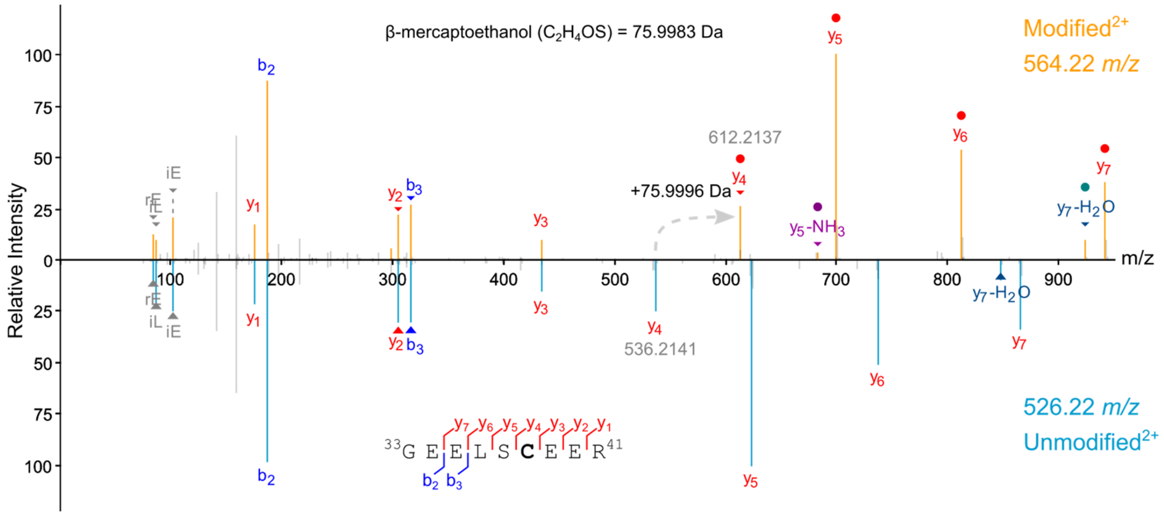

Figure S2 | MS/MS spectra identifying β-mercaptoethanol-modification on Cys38 of 14-3-3σ. Mirrored spectra for peptide [33]GEELS<u>C</u>EER[41] following tryptic digestion of 14-3-3σ. Comparing spectra for this peptide shows that the +76 Da modification observed on the intact 14-3-3σ monomer is localised to Cys38 and is likely to be β-mercaptoethanol capping of this cysteine. The following fragment ions are annotated, standard *b*/*y* type ions, neutral loss products (–NH_3 and –H_2O) and immonium/related ions (iE/L and rE).

Binding of FC-A to 14-3-3σ protein complexes

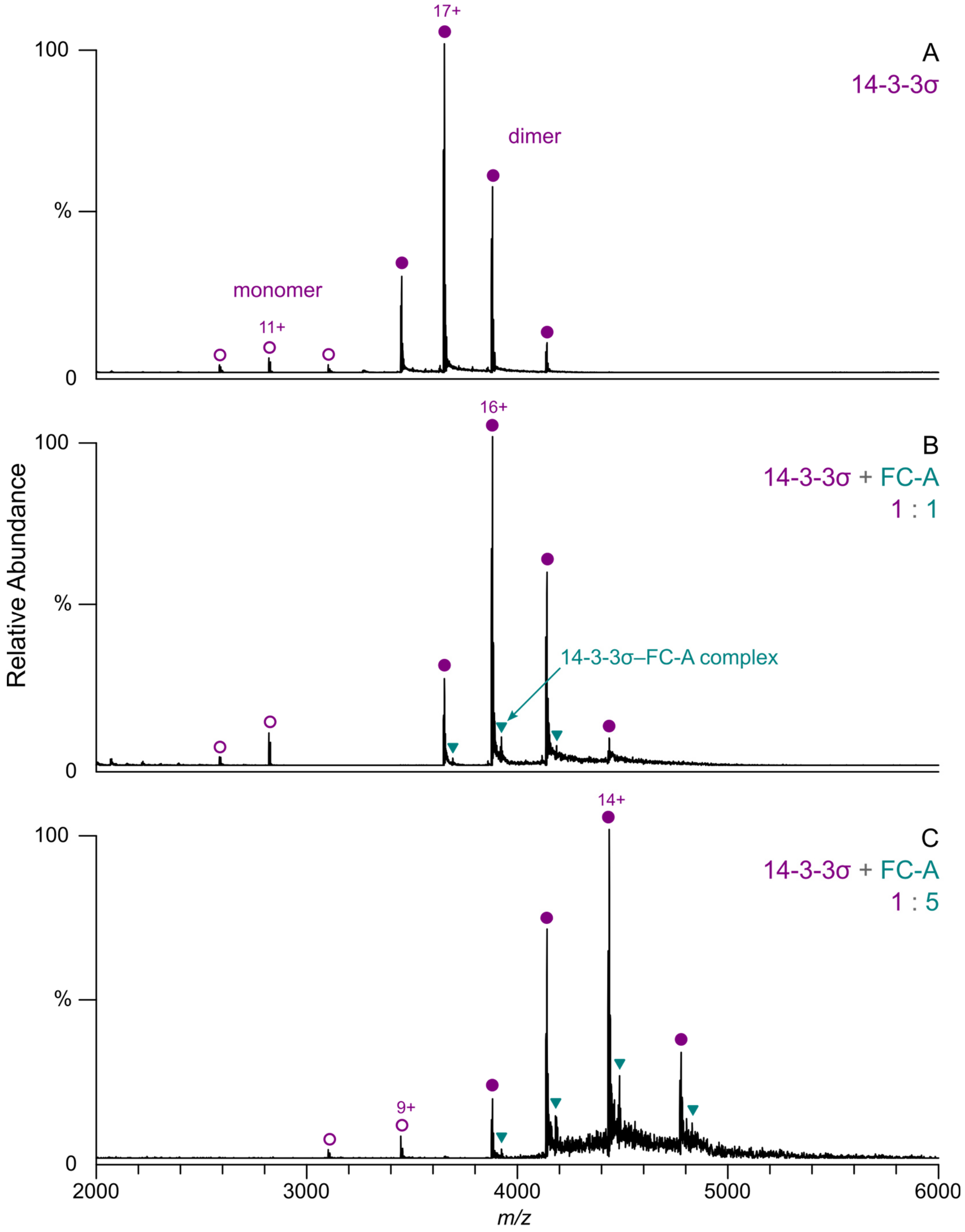

Figure S3 | Native mass spectra of FC-A binding to 14-3-3σ at 1:1 and 1:5 ratios. Sprayed from 50 mM ammonium acetate, 0.25% DMSO. **A**) 14-3-3σ (5 µM) only. **B**) 14-3-3σ (5 µM) with FC-A (5 µM) showing complex formation (~8% bound FC-A) and a slight charge shift (16+ most abundant). **C**) 14-3-3σ (5 µM) with FC-A (25 µM) showing complex formation (~15% bound FC-A) and a further charge shift (14+ most abundant).

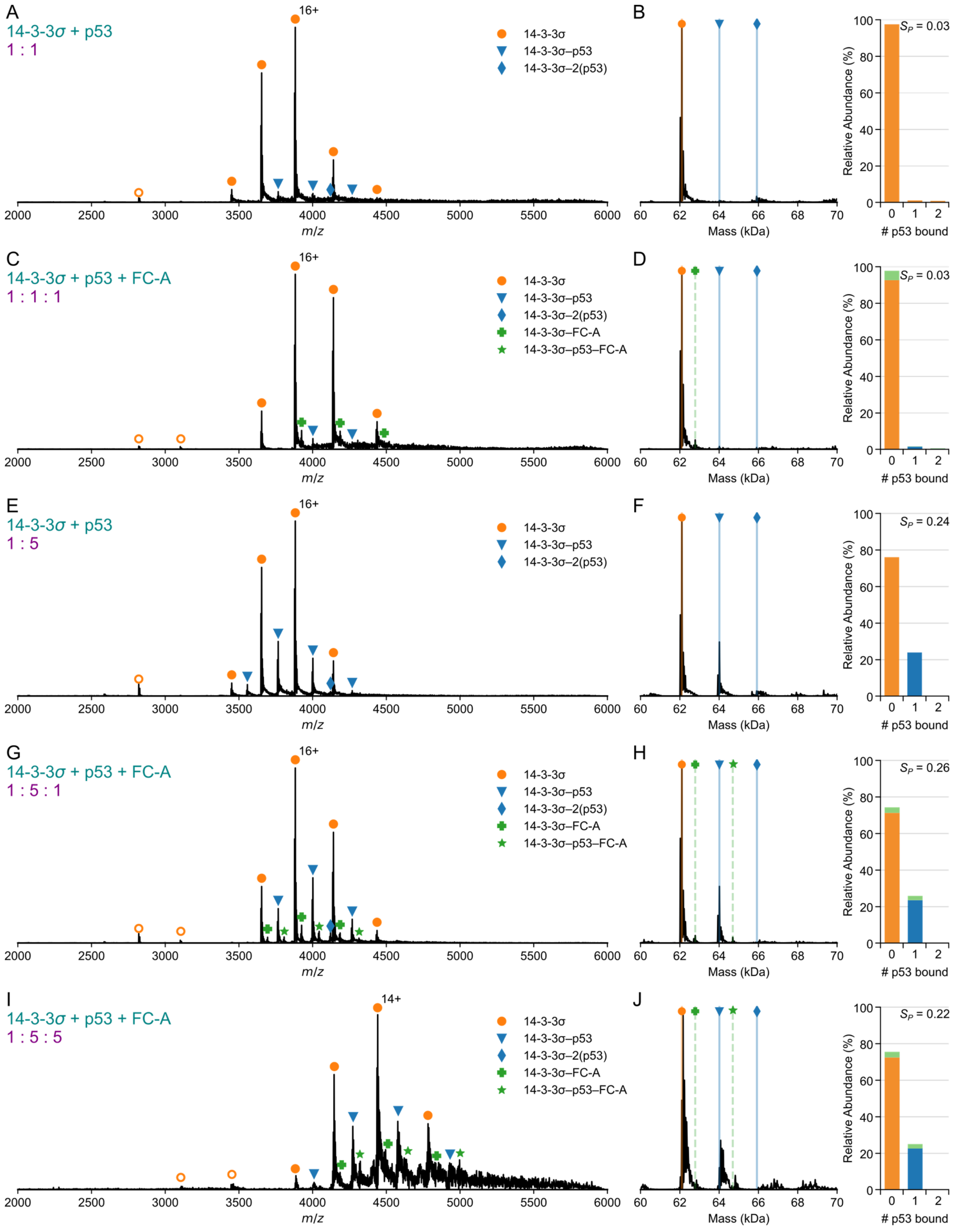

Figure S4 | Native mass spectra of 14-3-3σ, p53 and FC-A at different ratios. Binding of 14-3-3σ (5 µM) mixed with p53 (5 µM: **A–D** or 25 µM: **E–J**) in the absence (**A/B** and **E/F**) and presence of FC-A (5 µM: **C/D** and **G/H** or 25 µM: **I/J**). Mass spectra (**A**, **C**, **E**, **G**, **I**) and deconvoluted zero-charge mass spectra and bar charts (**B**, **D**, **F**, **H**, **J**) show moderate binding of p53 to 14-3-3σ with some low binding of FC-A. Binding of FC-A does not alter the proportion of bound p53. Mixed stoichiometries are annotated with shapes as follows: 14-3-3σ (orange circle), 14-3-3σ–p53 (blue triangle), 14-3-3σ–2(p53) (blue diamond), 14-3-3σ–FC-A (green plus), 14-3-3σ–p53–FC-A (green star) and 14-3-3σ–2(p53)–FC-A (pink pentagon). Dimer species are annotated with filled shapes while monomer species are hollow shapes. Lines are indicated on the deconvoluted spectra for expected masses of these stoichiometries.

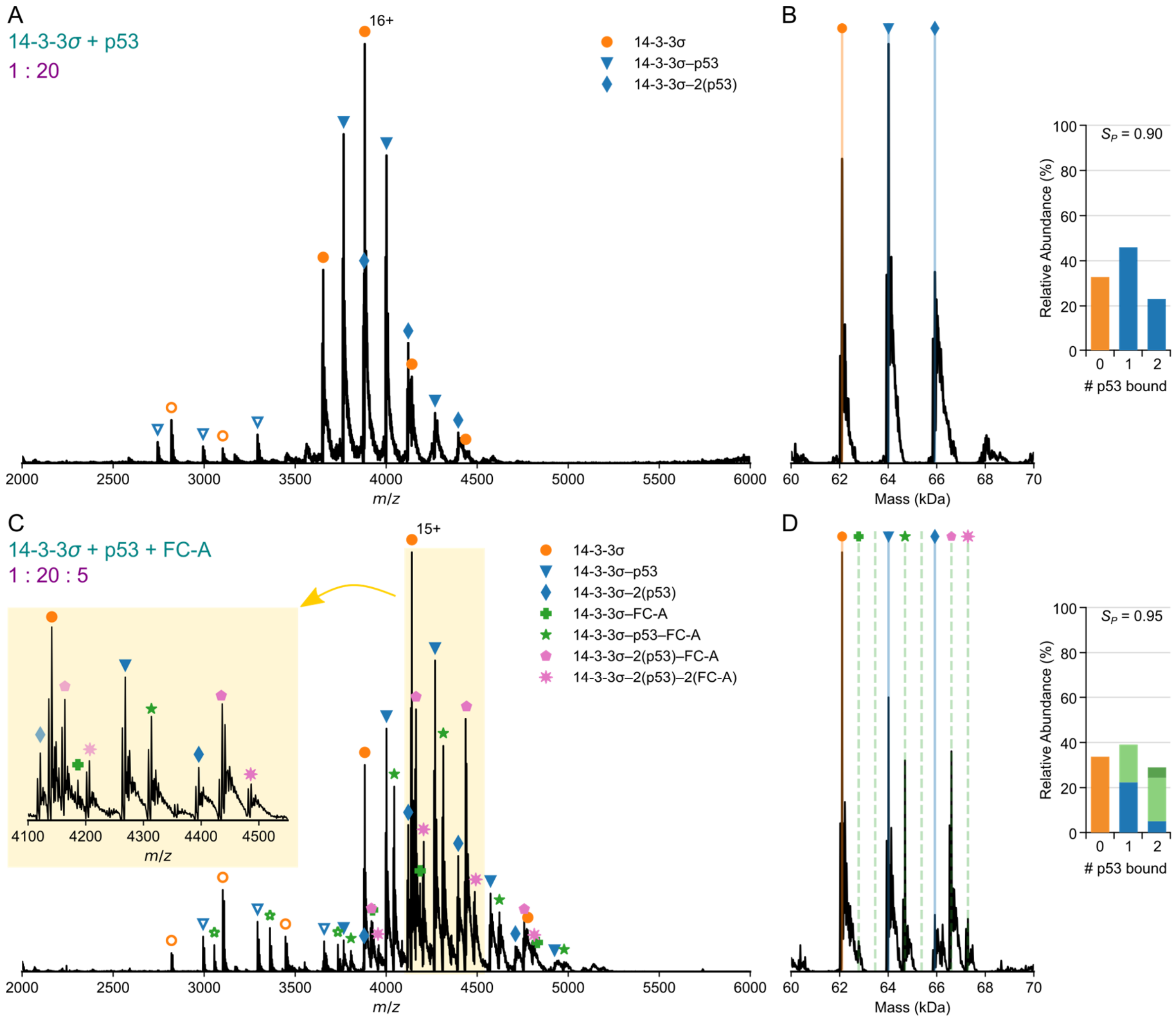

Figure S5 | Native mass spectra of 14-3-3σ, p53 and FC-A at significant excess. Binding of 14-3-3σ (5 µM) mixed with p53 (100 µM) in the absence (**A**/**B**) and presence of FC-A (25 µM: **C**/**D**). Mass spectra (**A**, **C**) and deconvoluted zero-charge mass spectra and bar charts (**B**, **D**) show significant binding of p53 to 14-3-3σ. Binding of FC-A only slightly alters the binding of p53, however, the presence of bound p53 seems to increase the binding of FC-A, this is particularly apparent in the 14-3-3σ–2(p53) species where a majority of this species is FC-A bound. This is contrast to ERα stabilisation (see Figure S6) where the binding of FC-A appears to promote ERα binding with shifts in S_P, even at low concentrations, and complete ERα–FC-A binding. A static gold-coated borosilicate nanospray emitter was used for these spectra, rather than a NanoMate due to the difficulty in spraying such high concentrations of peptide and FC-A. All instrument conditions were the same as used in all other experiments except capillary voltage which was held at 1 kV with a mass range of 2000–6000 *m/z*. Mixed stoichiometries are annotated with shapes as follows: 14-3-3σ (orange circle), 14-3-3σ–p53 (blue triangle), 14-3-3σ–2(p53) (blue diamond), 14-3-3σ–FC-A (green plus), 14-3-3σ–p53–FC-A (green star), 14-3-3σ–2(p53)–FC-A (pink pentagon) and 14-3-3σ–2(p53)–2(FC-A) (pink eight star). Dimer species are annotated with filled shapes while monomer species are hollow shapes. Lines are indicated on the deconvoluted spectra for expected masses of these stoichiometries.

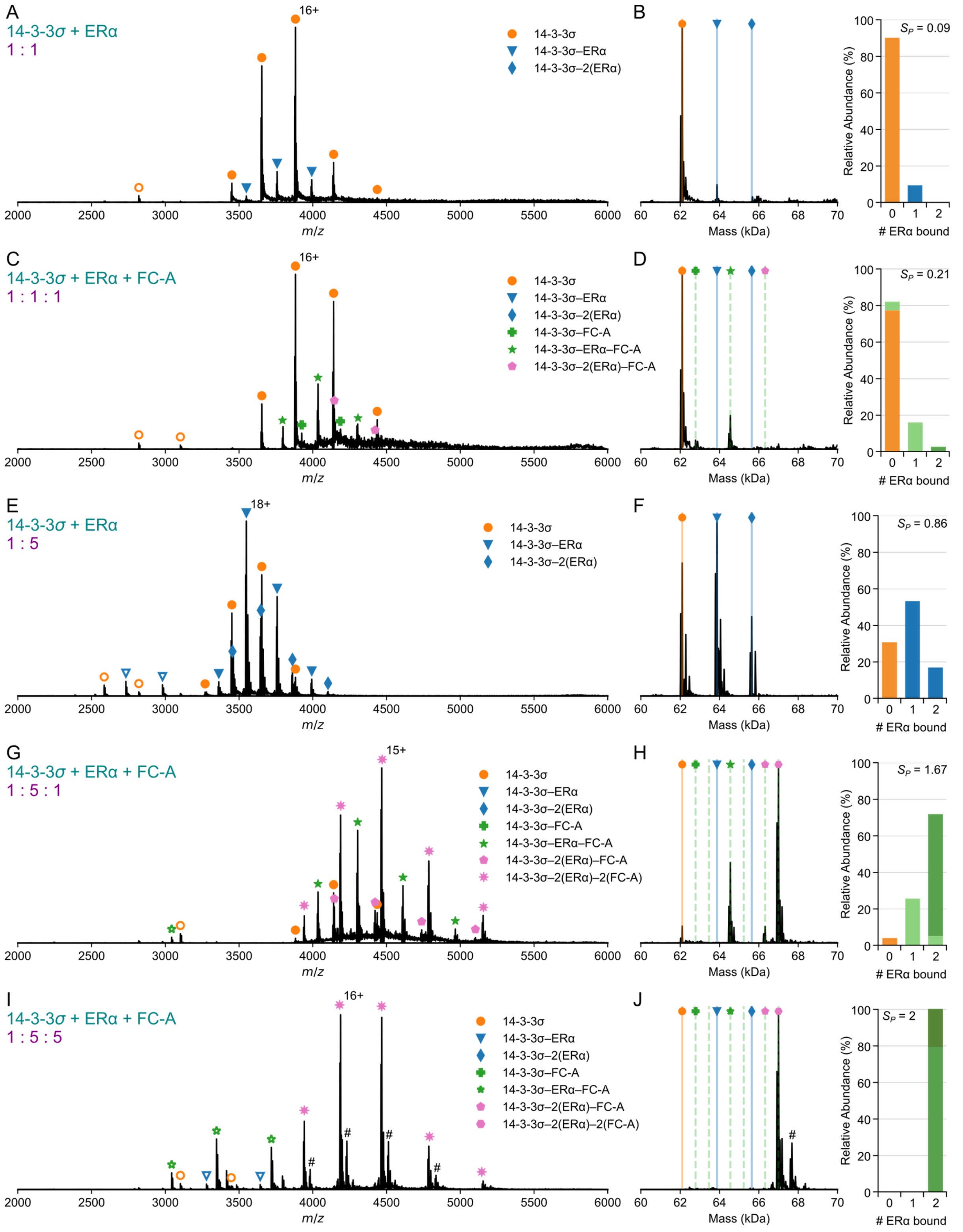

Figure S6 | Native mass spectra of 14-3-3σ, ERα and FC-A at different ratios. Binding of 14-3-3σ (5 µM) mixed with ERα (5 µM: **A–D** or 25 µM: **E–J**) in the absence (**A**/**B** and **E**/**F**) and presence of FC-A (5 µM: **C**/**D** and **G**/**H** or 25 µM: **I**/**J**). Mass spectra (**A**, **C**, **E**, **G**, **I**) and deconvoluted zero-charge mass spectra and bar charts (**B**, **D**, **F**, **H**, **J**) show significant binding of ERα to 14-3-3σ. Binding of FC-A significantly shifts the stoichiometry of the complexes to 2(14-3-3σ)–2(ERα)–2(FC-A) with negligible unbound 14-3-3σ. Binding of FC-A does not alter the proportion of bound p53. Mixed stoichiometries are annotated with shapes as follows: 14-3-3σ (orange circle), 14-3-3σ–ERα (blue triangle), 14-3-3σ–2(ERα) (blue diamond), 14-3-3σ–FC-A (green plus), 14-3-3σ–ERα–FC-A (green star), 14-3-3σ–2(ERα)–FC-A (pink pentagon) and 14-3-3σ–2(ERα)–2(FC-A) (pink eight star). Dimer species are annotated with filled shapes while monomer species are hollow shapes. Lines are indicated on the deconvoluted spectra for expected masses of these stoichiometries.

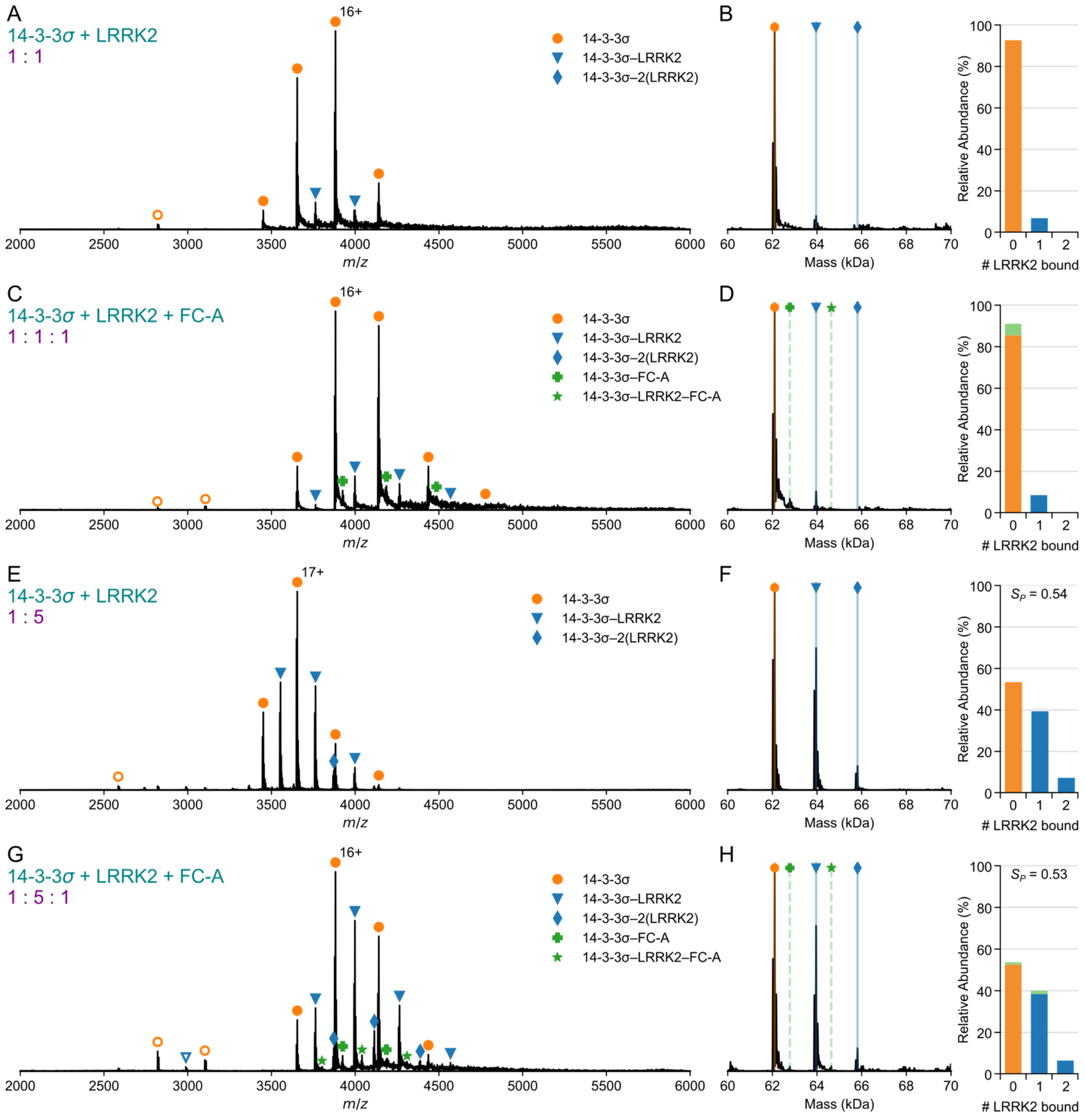

Figure S7 | Native mass spectra of 14-3-3σ, LRRK2 and FC-A at different ratios. Binding of 14-3-3σ (5 µM) mixed with LRRK2 (5 µM: **A–D** or 25 µM: **E–H**) in the absence (**A**/**B** and **E**/**F**) and presence of FC-A (5 µM: **C**/**D** and **G**/**H**). Mass spectra (**A**, **C**, **E**, **G**) and deconvoluted zero-charge mass spectra (**B**, **D**, **F**, **H**) show significant binding of LRRK2 to 14-3-3σ with some low binding of FC-A. Binding of FC-A reduces the overall binding of LRRK2 to 14-3-3σ. Mixed stoichiometries are annotated with shapes as follows: 14-3-3σ (orange circle), 14-3-3σ–LRRK2 (blue triangle), 14-3-3σ–2(LRRK2) (blue diamond), 14-3-3σ–FC-A (green plus) and 14-3-3σ–LRRK2–FC-A (green star). Dimer species are annotated with filled shapes while monomer species are hollow shapes. Lines are indicated on the deconvoluted spectra for expected masses of these stoichiometries.

Binding of drug cocktail to 14-3-3σ protein complexes

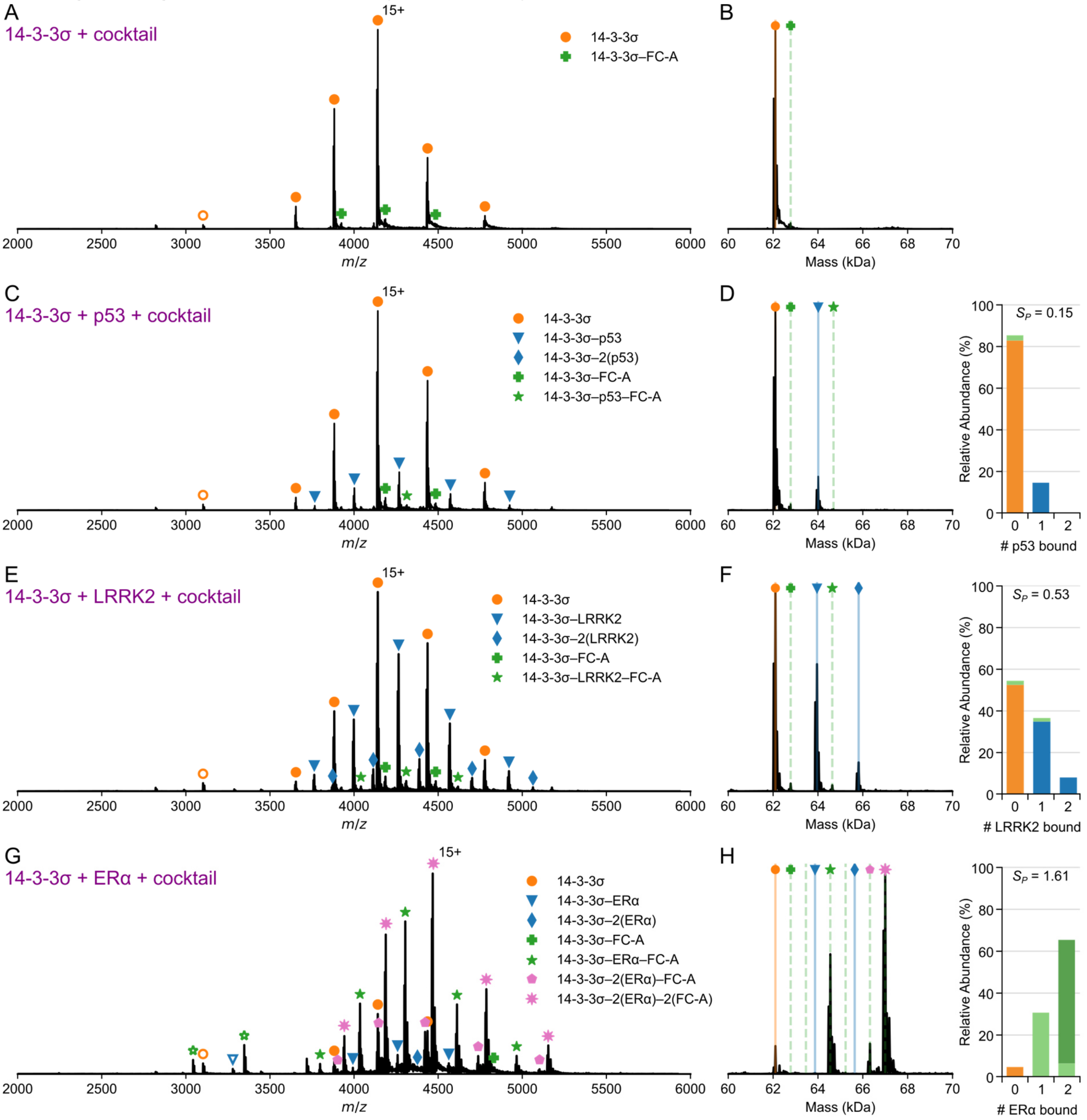

Figure S8 | Differential binding of drug cocktail to 14-3-3σ protein complexes with p53, LRRK2 and ERα. Binding of 14-3-3σ (5 µM) mixed with drug cocktail (5 µM each) and peptides: p53 (**C**/**D**), LRRK2 (**E**/**F**) and ERα (**G**/**H**). Drug cocktail components were FC-A (5 µM), Epi (5 µM), Res (5 µM), Bez (5 µM) and Dan (5 µM); 0.25% DMSO, final concentration. Mass spectra (**A**, **C**, **E**, **G**) and deconvoluted zero-charge mass spectra (**B**, **D**, **F**, **H**) show reduced binding of FC-A to 14-3-3σ (c.f. Figure S3) and no other cocktail components are seen to bind (see Figure S9 for mixing of individual drugs with 14-3-3σ/ERα). The binding stoichiometry of 14-3-3σ/ERα complexes is less pronounced than with FC-A alone (see Figure S6) yet still shows prominent stabilisation. Mixed stoichiometries for each partner (p53, LRRK2 or ERα) are annotated with shapes as follows: 14-3-3σ (orange circle), 14-3-3σ–partner (blue triangle), 14-3-3σ–2(partner) (blue diamond), 14-3-3σ–FC-A (green plus), 14-3-3σ–partner–FC-A (green star), 14-3-3σ–2(partner)–FC-A (pink pentagon) and 14-3-3σ–2(partner)–2(FC-A) (pink eight star). Dimer species are annotated with filled shapes while monomer species are hollow shapes. Lines are indicated on the deconvoluted spectra for expected masses of these stoichiometries.

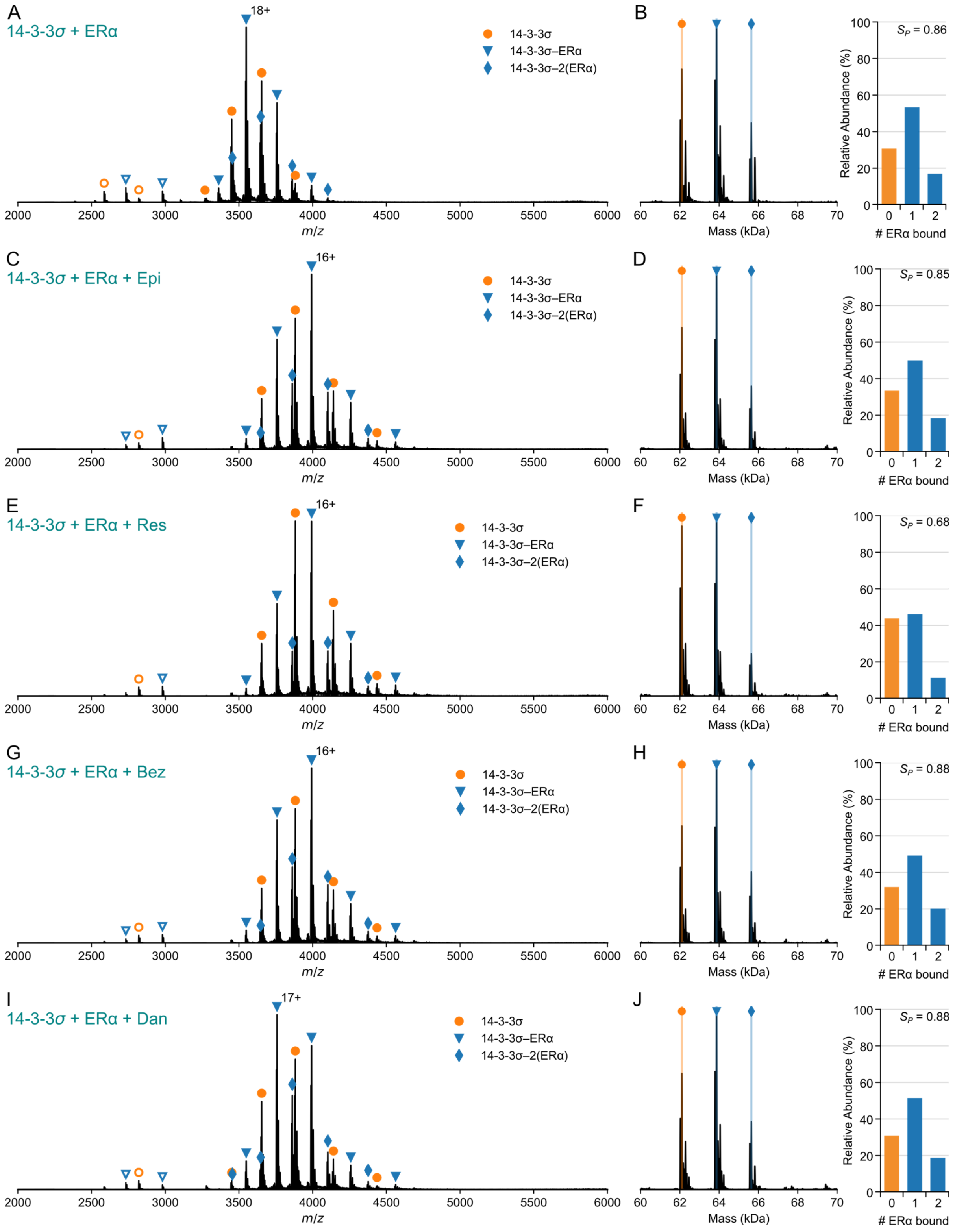

Figure S9 | Negligible binding of drugs: Epi, Res, Bez and Dan to 14-3-3σ–ERα protein complexes at a 1:5:1 ratio. Binding of 14-3-3σ (5 µM) mixed with ERα (25 µM) and drugs (5 µM each). Mass spectra (**A**, **C**, **E**, **G**, **I**) and deconvoluted zero-charge mass spectra (**B**, **D**, **F**, **H**, **J**) show negligible binding of selected drugs to 14-3-3σ protein complexes. All drugs cause a decrease in average charge state. While no binding is observed, Res causes an increase in the unbound 14-3-3σ. 14-3-3σ (orange circle), 14-3-3σ–ERα (blue triangle), 14-3-3σ–2(ERα) (blue diamond). Dimer species are annotated with filled shapes while monomer species are hollow shapes.

Nonspecific binding of phosphopeptides and drug compounds

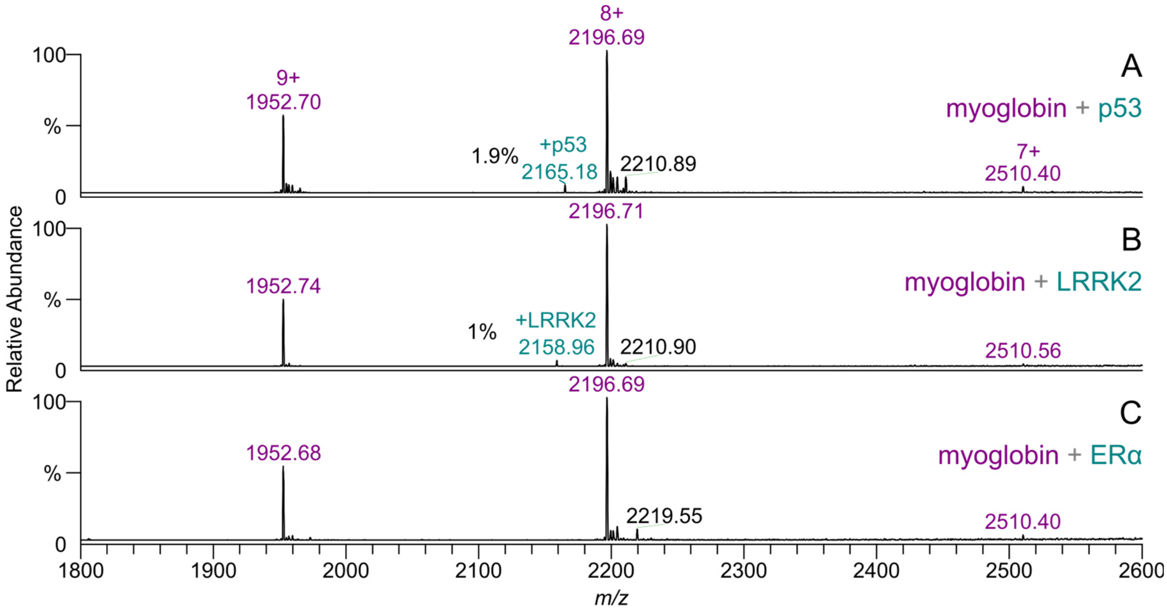

Figure S10 | Negligible non-specific binding of p53, LRRK2 and ERα to myoglobin. A) myoglobin (3 µM) mixed with p53 (25 µM) shows low binding of p53 to the 9+ charge state (2165 *m/z*). **B**) myoglobin (3 µM) mixed with LRRK2 (25 µM) shows low binding of LRRK2 to the 9+ charge state (2159 *m/z*). **C**) myoglobin (3 µM) mixed with ERα (25 µM) shows no binding of ERα.

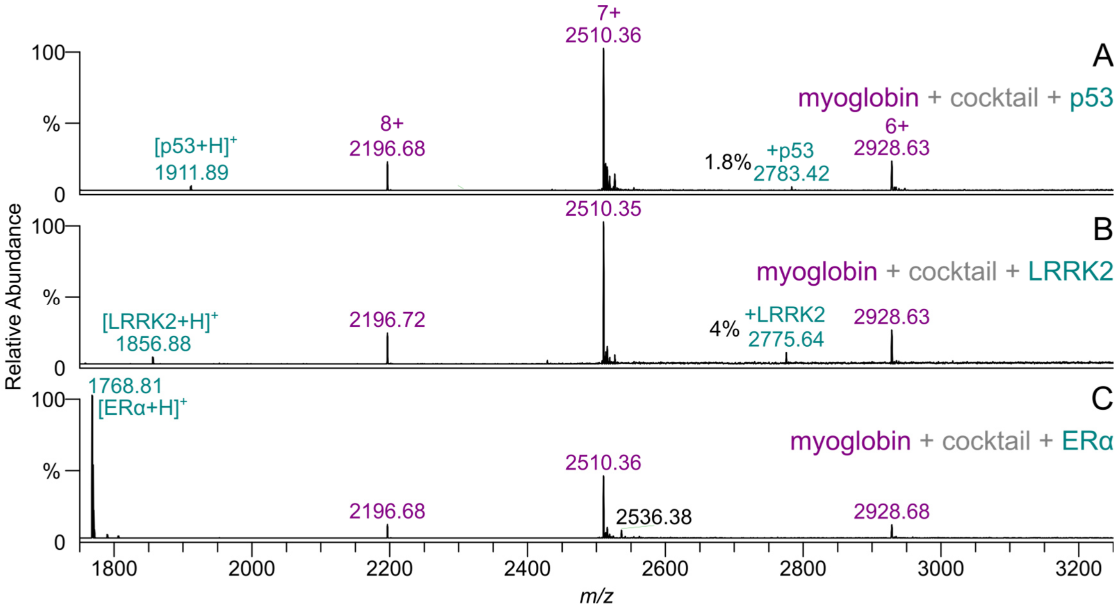

Figure S11 | Negligible non-specific binding of p53, LRRK2 and ERα, and drug cocktail to myoglobin. Charge state shift, compared to Figure S10, is seen, with 7+ the most abundant. Ions corresponding to 1+ phosphopeptides are also seen. Drug cocktail components: FC-A (5 µM), Epi (5 µM), Res (5 µM), Bez (5 µM) and Dan (5 µM). **A**) myoglobin (3 µM) mixed with p53 (25 µM) and cocktail shows low binding of p53 to the 7+ charge state (2783 *m/z*). **B**) myoglobin (3 µM) mixed with LRRK2 (25 µM) and cocktail shows low binding of LRRK2 to the 7+ charge state (2776 *m/z*). **C**) myoglobin (3 µM) mixed with ERα (25 µM) and cocktail shows no binding of ERα.

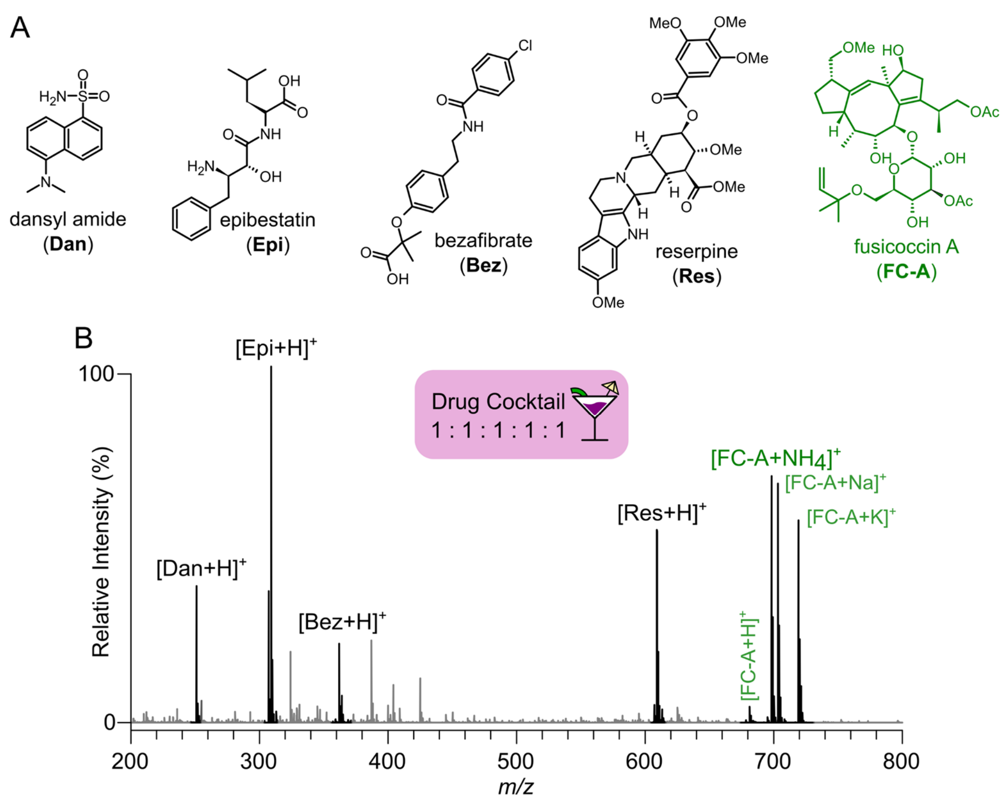

Figure S12 | Spectra of drug cocktail (Dan, Epi, Bez, Res, FC-A). Drug cocktail components were FC-A (5 µM), Epi (5 µM), Res (5 µM), Bez (5 µM) and Dan (5 µM); 0.25% DMSO, final concentration. **A**) Skeletal structures of drugs in cocktail. **B**) Spectrum of mass region containing singly charged drugs.

Screening extended drug cocktail for 14-3-3σ–ERα stabilisers

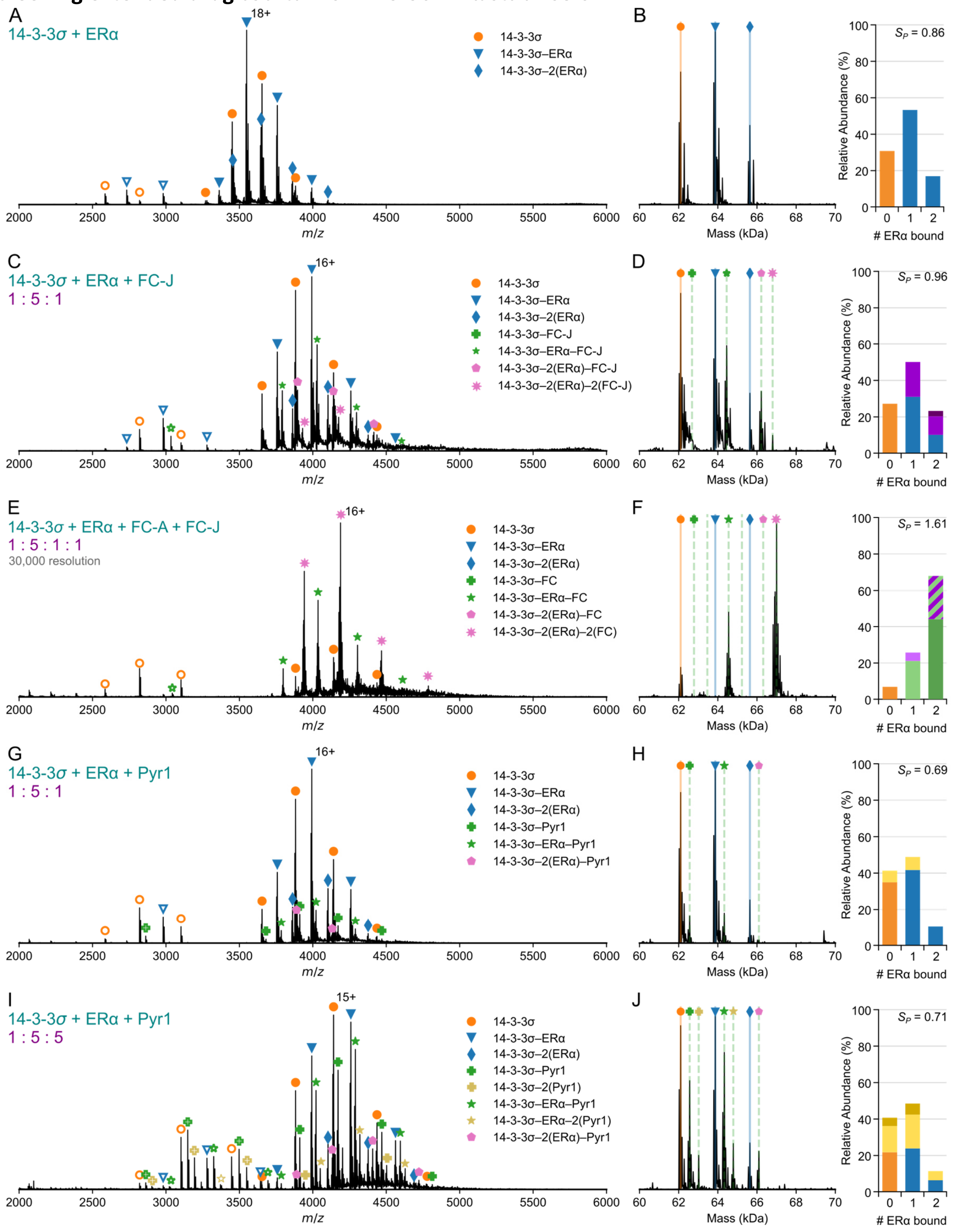

Figure S13 | Native mass spectra of 14-3-3σ and ERα binding to FC-J or Pyr1. Binding of 14-3-3σ (5 µM) mixed with ERα (5 µM) in the absence (**A**/**B**) and presence of FC-J (5 µM: **C**/**D**), FC-A and FC-J (5 µM each; **E**/**F**) or Pyr1 (5 µM; **G**/**H** of 25 µM; **I**/**J**). Note: Pyr1 concentrations given are of the active *R* enantiomer as the synthesised Pyr1 is racemic. Mass spectra (**A**, **C**, **E**, **G**, **I**) and deconvoluted zero-charge mass spectra and bar charts (**B**, **D**, **F**, **H**, **J**) show significant binding of ERα to 14-3-3σ. Mixture containing FC-A and FC-J (**E**/**F**) was recorded at a mass resolution of 30,000 to reduce overlap between β-mercaptoethanol-capped variants. FC-A and FC-J were observed to form mixed dimers at 1183.72 *m/z* (*A*–*A*), 1283.73 *m/z* (*A*–*J*) and 1383.75 *m/z* (*J*–*J*). Mixed stoichiometries are annotated with shapes as follows: 14-3-3σ (orange circle), 14-3-3σ–ERα (blue triangle), 14-3-3σ–2(ERα) (blue diamond), 14-3-3σ–drug (green plus), 14-3-3σ–ERα–drug (green star), 14-3-3σ–2(ERα)–drug (pink pentagon) and 14-3-3σ–2(ERα)–2(drug) (pink eight star). Dimer species are annotated with filled shapes while monomer species are hollow shapes. Lines are indicated on the deconvoluted spectra for expected masses of these stoichiometries.

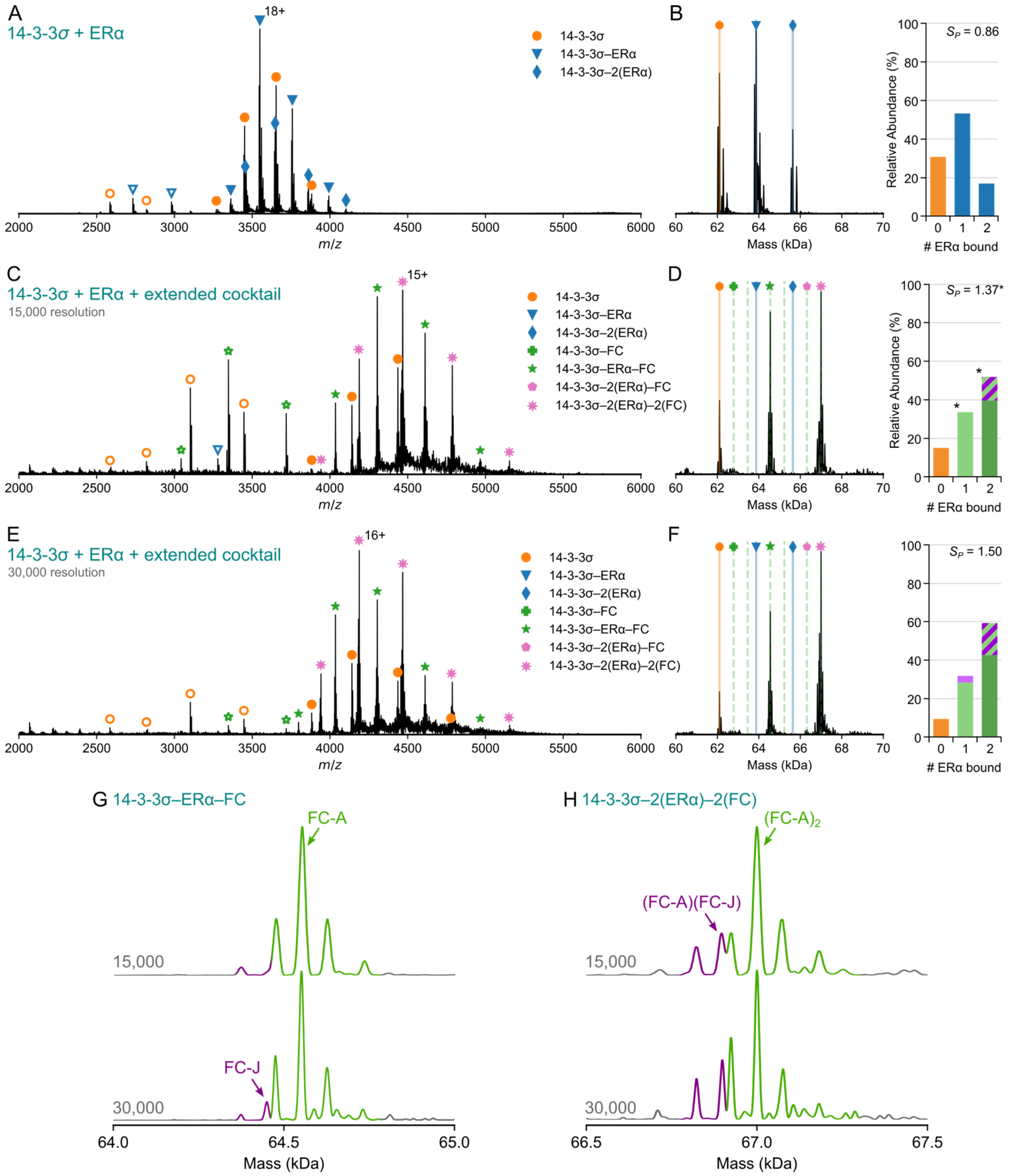

Figure S14 | Extended cocktail binding to 14-3-3σ and ERα. Binding of 14-3-3σ (5 µM) mixed with ERα (5 µM) in the absence (**A**/**B**) and presence of the extended cocktail (**C**–**F**). Spectra recorded at 15,000 resolution (**A**–**D**, **G**–**H**) and 30,000 resolution (**E**–**H**) show that overlap of FC-A and FC-J bound to 14-3-3σ–ERα can be mitigated—this is only necessary due to the variable β-mercaptoethanol modification of this recombinant 14-3-3σ. Drug cocktail components were FC-A (5 µM), FC-J (5 µM), Pyr1 (5 µM), Epi (5 µM), Res (5 µM), Bez (5 µM) and Dan (5 µM); 0.35% DMSO, final concentration. Note: Pyr1 concentrations given are of the active *R* enantiomer as the synthesised Pyr1 is racemic. Mass spectra (**A**, **C**, **E**) and deconvoluted zero-charge mass spectra and bar charts (**B**, **D**, **F**, **G**, **H**) show binding of both FC-A and FC-J to 14-3-3σ–ERα to different extents with some co-occupancy of 14-3-3σ–2(ERα) by both drugs; negligible $(FC\text{-}J)_2$ was observed. Close-ups of deconvoluted spectra for the 14-3-3σ–ERα–FC (G) and 14-3-3σ–2(ERα)–2(FC) (H) regions of the extended cocktail at 15,000 and 30,000 mass resolution show that higher resolution is necessary to appropriately assign and integrate the different stoichiometries are report the correctly determined S_P values (* in C). Mixed stoichiometries are annotated with shapes as follows: 14-3-3σ (orange circle), 14-3-3σ–ERα (blue triangle), 14-3-3σ–2(ERα) (blue diamond), 14-3-3σ–FC (green plus), 14-3-3σ–ERα–FC (green star), 14-3-3σ–2(ERα)–FC (pink pentagon) and 14-3-3σ–2(ERα)–2(FC) (pink eight star). Dimer species are annotated with filled shapes while monomer species are hollow shapes. Lines are indicated on the deconvoluted spectra for expected masses of these stoichiometries.

Table S1 | Molecular weight assignments for protein, peptides and drug compounds. The theoretical and observed molecular weights for 14-3-3σ, p53, LRRK2, ERα, FC-A, Epi, Res, Bez and Dan. All masses are given as neutral masses, and were determined from either $[M + nH]^{n+}$ peaks (local maxima) or as differences between adjacent peaks. [a] Calculated from amino acid sequences using ExPASy ProtParam. [b] Calculated from elemental composition. [c] Calculated from difference between adjacent peaks (bound and unbound) of the 16+ charge state of 14-3-3σ dimer.

Protein/Peptide/ Complex	Notes	Theoretical Mass (avg.)	Theoretical Mass (mono)	Observed Mass (avg.)	Observed Mass (mono)
**14-3-3σ (A)**	– N-Met	31014.5 [a]	—	31013 ± 0.8	—
**14-3-3σ (B)**	– N-Met + β-ME	31090.6 [a]	—	31089 ± 1.2	—
**14-3-3σ dimer**	A + A	62029.0 [a]	—	62026 ± 0.4	—
**14-3-3σ dimer**	A + B	62105.1 [a]	—	62102 ± 0.5	—
**14-3-3σ dimer**	B + B	62181.2 [a]	—	62174 ± 0.7	—
**p53**	$C_{79}H_{128}N_{23}O_{28}PS$	1911.04 [b]	1909.88 [b]	1911.04 [c]	1909.89
**LRRK2**	$C_{77}H_{119}N_{26}O_{26}P$	1855.91 [b]	1854.85 [b]	1855.68 [c]	1854.87
**ERα**	$C_{77}H_{119}N_{26}O_{26}P$	1767.83 [b]	1766.78 [b]	1767.59 [c]	1766.81
**FC-A**	$C_{36}H_{56}O_{12}$	680.82 [b]	680.377 [b]	680.96 [c]	680.378
**Epi**	$C_{16}H_{24}N_2O_4$	308.37 [b]	308.174 [b]	—	308.173
**Res**	$C_{33}H_{40}N_2O_9$	608.68 [b]	608.273 [b]	—	608.274
**Bez**	$C_{19}H_{20}ClNO_4$	361.82 [b]	361.108 [b]	—	361.108
**Dan**	$C_{12}H_{14}N_2O_2S$	250.32 [b]	250.078 [b]	—	250.078
**FC-J**	$C_{32}H_{52}O_9$	580.75 [b]	580.361 [b]	580.96 [c]	580.362
**Pyr1**	$C_{24}H_{16}N_2O_8$	460.39 [b]	460.091 [b]	460.48 [c]	460.091

Synthesis of pyrrolidone 1 (Pyr1)

Pyr1 was prepared using the synthetic route illustrated in Scheme S1 which was adapted from a literature report.[1]

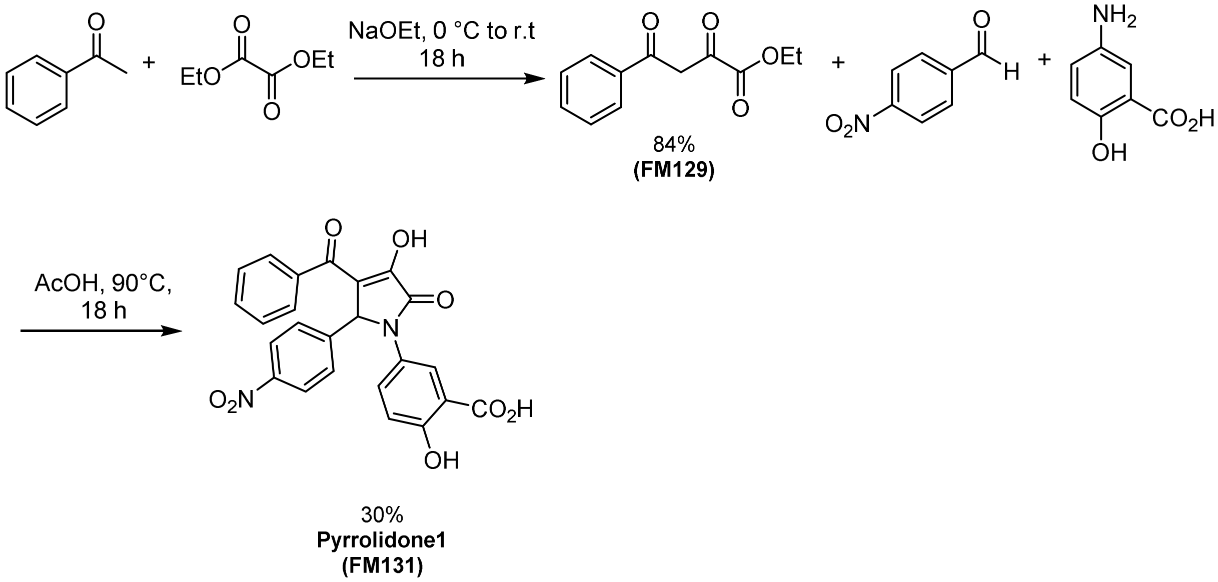

Scheme S1 | Synthetic route to Pyr1. Synthetic route to (±)-(5-(3-benzoyl-4-hydroxy-2-(4-nitrophenyl)-5-oxo-2,5-dihydro-1H-pyrrol-1-yl)-2-hydroxybenzoic acid (Pyr1, FM131).

General experimental notes: Reactions were carried out under an inert N_2 atmosphere unless otherwise specified. Reagents and solvents used in all reactions were purchased from Sigma Aldrich, Alfa Aesar or Fisher Scientific and used without further purification. Nuclear Magnetic Resonance (NMR) spectra were collected at 25 °C using a Bruker 500 instrument (^1H NMR; 500 MHz, ^{13}C NMR; 125 MHz) with chemical shifts given in parts per million (ppm). Samples were run in $CDCl_3$ or DMSO-d_6. ^1H NMR data has been reported as: chemical shift (δ), (proton integration, multiplicity, coupling constant (Hz), assignment). Multiplicities have been reported as: s = singlet, d = doublet, dd = doublet of doublets, t = triplet, and m = multiplet. MestReNova 6.0.2 software was used to analyse the spectra. High resolution electrospray ionisation mass spectroscopy was performed on a Waters Acquity XEVO Q ToF instrument and the data analysed using MassLynx. HPLC analysis was performed on a ThermoFisher Ultimate3000 system with Chromeleon software using a reverse phase Phenomenex Luna® 5 µm C18 100 Å, LC Column (250 x 21.2 mm) using the following method (solvent A = 0.1% TFA in H_2O, solvent B = 0.1%TFA in MeCN): 5% B for 5 min, 5-100% B over 30 min, 100% B for 5 min, 100-10% B for 5 min, 10% B for 5 min at 0.5 ml/min.

Ethyl-2,4-dioxo-4-phenylbutanonate ***(FM129)***

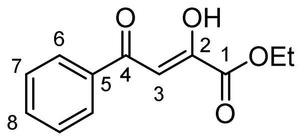

FM129

To a solution of 1.00 M sodium ethoxide (21% in EtOH, 4.57 mL, 4.60 mmol, 1.1 eq) was added acetophenone (0.485 mL, 4.20 mmol, 1.00 eq) followed by diethyl oxalate (0.563 mL, 4.20 mmol, 1.00 eq) at 0 °C under N_2. The resulting suspension was stirred for 18 h at room temperature. The reaction mixture was quenched with a aqueous solution of HCl (10 mL, 1.0 M) until pH=6. The organic phase was separated and the aqueous phase was further extracted with EtOAc (3 x 20 mL). The combined organic phase was washed with water (20 mL), dried (Na_2SO_4), filtered and concentrated *in vacuo* to furnish **FM129** (770 mg, 84%) as an orange-brown powder. *Rf* = 0.22 (4:1 Hex:EtOAc). ^1H-NMR (500 MHz, $CDCl_3$): δ (ppm) 15.23 (1H, broad s, O*H*), 7.92 (2 H, d, *J* 7.6,

H-6), 7.54 (1 H, app. t, *J* 7.4, H-8), 7.44 (2 H, app. t, *J* 7.6, H-7), 7.01 (1 H, s, H-3), 4.34 (2 H, q, *J* 7.1, OC*H*$_2$CH$_3$), 1.35 (3 H, t, *J* 7.1, *OCH$_2$CH$_3$*). ^{13}C NMR (125 MHz, CDCl$_3$): δ (ppm) 190.8 (C-2), 169.8 (C-4), 162.2 (C-1), 134.9 (C-5), 133.8 (C-8), 128.9 (C-7), 127.9 (C-6), 97.9 (C-3), 62.6 (O*C*H$_2$CH$_3$), 14.1 (OCH$_2$*C*H$_3$). HRMS (ESI): m/z [M-H]$^-$ calc. for $C_{12}H_{11}O_4$: 219.0657, observed: 219.0658.

5-(3-Benzoyl-4-hydroxy-2-(4-nitrophenyl)-5-oxo-2,5-dihydro-1H-pyrrol-1-yl)-2-hydroxybenzoic acid **(Pyr1, FM131)**.

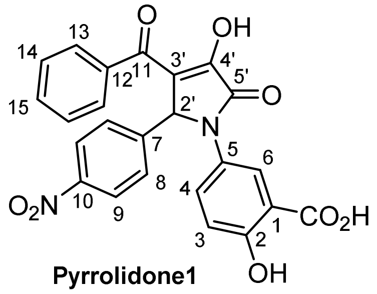

Pyrrolidone1 (FM131)

To a suspension of **FM129** (200 mg, 0.90 mmol, 1.00 eq) in acetic acid (15.0 mL) was added 4-nitrobenzaldehyde (137.0 mg, 0.90 mmol, 1.00 eq) followed by 2-amino-5-hydroxybenzoic acid (139.0 mg, 0.90 mmol, 1.00 eq). The reaction mixture was heated at 90 °C for 18 h. After cooling to room temperature, the mixture was diluted with Et$_2$O (10.0 mL) and filtered. The residue was washed with Et$_2$O and dried to furnish **FM131** (140.0 mg, 30% yield) as a colourless powder. *Rf* = 0.07 (9:1 DCM/MeOH). ^{1}H NMR (500 MHz, DMSO-d$_6$): δ (ppm) 8.05 (2 H, d, *J* 8.8, H-9), 8.03 (1 H, d, H 2.8, H-6), 7.73-7.68 (5 H, m, H-8, 13, 4), 7.58 (1 H, app. tt, *J* 7.2, 1.2, H-15), 7.45 (2 H, app. t, *J* 7.8, H-14), 7.03 (1 H, d, *J* 8.9, H-3), 6.58 (1 H, s, H-2'). ^{13}C NMR (126 MHz, DMSO): δ (ppm) 189.45 (C-11), 171.6 (C-4'), 165.0 (*C*OOH), 159.3 (C-2), 151.7 (C-5'), 147.7 (C-10), 144.9 (C-7), 138.4 (C-12), 133.1 (C-15), 130.9 (C-5), 129.7 (C-14), 129.19 (C-13), 128.6 (C-9), 127.9 (C-6), 125.40 (C-4), 124.0 (C-8), 119.5 (C-1), 118.0 (C-3), 113.7 (C-3'), 61.3(C-2'). HRMS (ESI): m/z [M+H]$^+$ calc. for $C_{24}H_{16}N_2O_8$: 461.0985, observed: 461.0987. HPLC retention time: 29.8 min (95.1% purity).

A

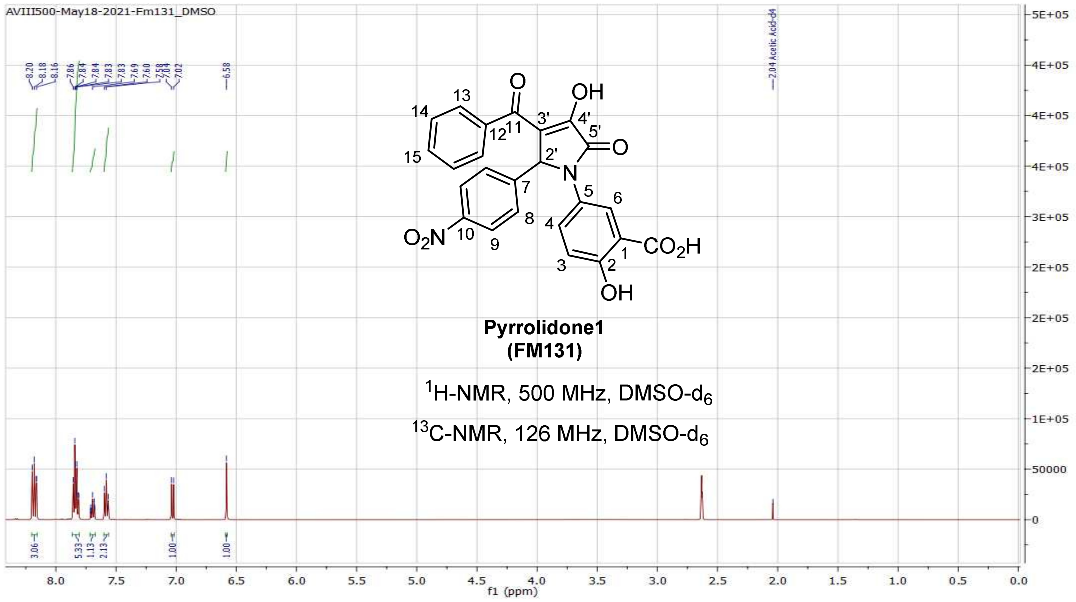

B

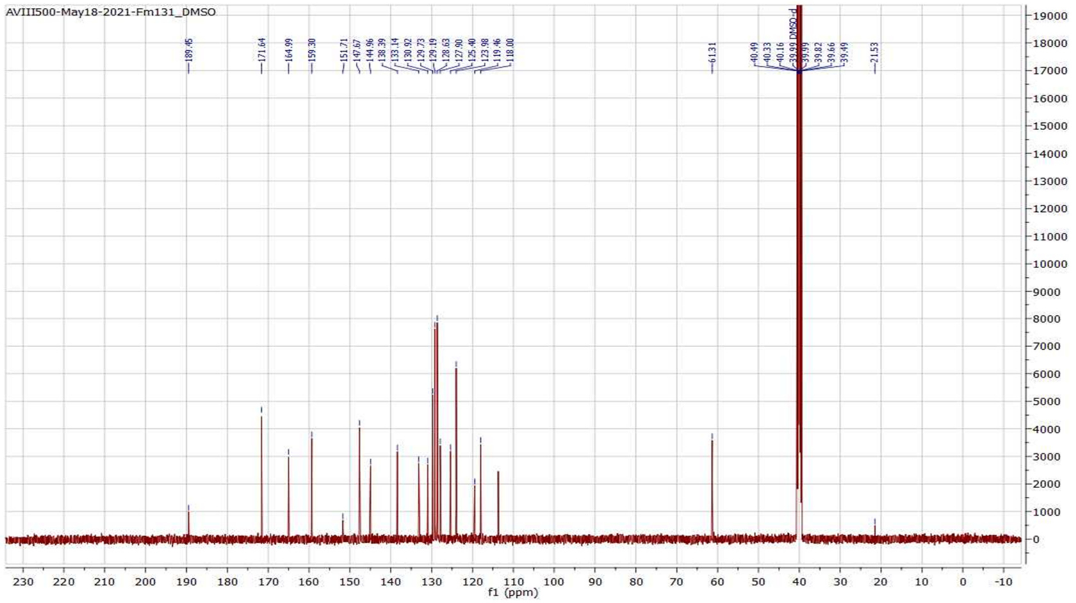

C

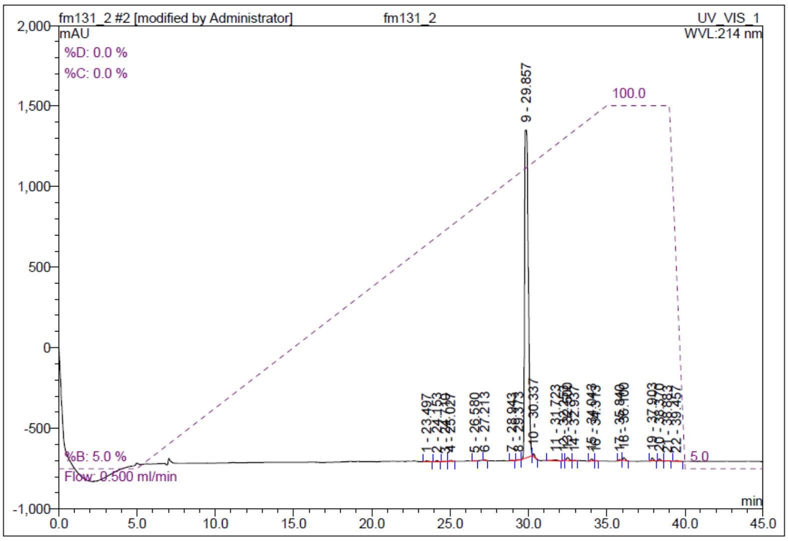

Figure S15 | Spectroscopic characterisation of Pyr1. A) 1H-NMR (500 MHz) in DMSO-d_6. **B**) 13C-NMR (126 MHz) in DMSO-d_6. **C**) HPLC-UV (214 nm). For assignments see previous section.

References

1 A. Richter, R. Rose, C. Hedberg, H. Waldmann and C. Ottmann, *Chem. – Eur. J.*, 2012, **18**, 6520–6527.
